# How Memory Conforms to Brain Development

**DOI:** 10.3389/fncom.2019.00022

**Published:** 2019-04-16

**Authors:** Ana P. Millán, Joaquín J. Torres, Joaquín Marro

**Affiliations:** Institute “Carlos I” for Theoretical and Computational Physics, University of Granada, Granada, Spain

**Keywords:** brain developing, brain structure and function, synaptic pruning, storage capacity, dynamic memories

## Abstract

Nature exhibits countless examples of *adaptive networks*, whose topology evolves constantly coupled with the activity due to its function. The brain is an illustrative example of a system in which a dynamic complex network develops by the generation and pruning of synaptic contacts between neurons while memories are acquired and consolidated. Here, we consider a recently proposed brain developing model to study how mechanisms responsible for the evolution of brain structure affect and are affected by memory storage processes. Following recent experimental observations, we assume that the basic rules for adding and removing synapses depend on local synaptic currents at the respective neurons in addition to global mechanisms depending on the mean connectivity. In this way a feedback loop between “form” and “function” spontaneously emerges that influences the ability of the system to optimally store and retrieve sensory information in patterns of brain activity or memories. In particular, we report here that, as a consequence of such a feedback-loop, oscillations in the activity of the system among the memorized patterns can occur, depending on parameters, reminding mind dynamical processes. Such oscillations have their origin in the destabilization of memory attractors due to the pruning dynamics, which induces a kind of structural disorder or noise in the system at a long-term scale. This constantly modifies the synaptic disorder induced by the interference among the many patterns of activity memorized in the system. Such new intriguing oscillatory behavior is to be associated only to long-term synaptic mechanisms during the network evolution dynamics, and it does not depend on short-term synaptic processes, as assumed in other studies, that are not present in our model.

## 1. Introduction

A complex interrelation between “form” and “function” is known to play an important role in nature (Gross and Blasius, 2008; Vazquez et al., 2008; Sayama et al., 2013). The idea has been efficiently developed in the field of *adaptive networks*, in which a sort of coupling feedback loop sets in between the network dynamic activity and its topological structure. Outstanding phenomena then emerge, including self-organization into complex topologies that exhibit robust dynamics, spontaneous differentiation of the nodes, or complex mutual dynamics in both activity and topology, in any case mimicking many different conditions in nature (Bullmore and Sporns, 2009; Sayama et al., 2013; Millán et al., 2018a). This framework has revealed quite useful to understand fundamental questions concerning mammal brains, e.g., how structural and functional properties relate to each other both at the level of models involving sets of neurons and synapses and at the coarse-grained scale of *connectomes* and functional nets which is captured by imaging techniques (Bullmore and Sporns, 2009). A main question that we can thus address is how an efficient brain develops by *synaptic pruning* after a sort of “wild” proliferation of synaptic connections between neurons following conception (Chechik et al., 1998; Iglesias et al., 2005; Santos and Noggle, 2011; Presumey et al., 2017). In humans, for example, synaptic density at birth is about twice that at puberty, and certain brain disorders, such as autism spectrum disorder (ASD) and schizophrenia, have been related to details of synaptic pruning (Keshavan et al., 1994; Geschwind and Levitt, 2007; Faludi and Mirnics, 2011; Kolb et al., 2012; Fornito et al., 2015). In particular, ASD has been associated with a defect of synaptic pruning in certain brain areas (Tang et al., 2014), whereas schizophrenia could be related to an excessive pruning (Sekar et al., 2016). In any case, it now seems clear that such synaptic pruning involves in some way an optimization process, probably aimed at minimizing both energy consumption and the genetic information that otherwise would be needed to build an efficient and robust network (Chechik et al., 1999; Chklovskii et al., 2004; Johnson et al., 2010; Knoblauch et al., 2010; Navlakha et al., 2015). In particular, recent studies on associative memory have shown that this process could greatly improve memory retrieval under a noisy environment, such as it is the case in biological systems (Millán et al., 2018a). Moreover, ongoing structural plasticity in the adult brain has also been suggested to improve substantially the storage capacity (Chklovskii et al., 2004; Knoblauch et al., 2010), and has been related to graded amnesia, catastrophic forgetting, and the spacing effect (Knoblauch et al., 2014; Knoblauch and Sommer, 2016). These results are based on the fact that the number of potential synapses a neuron could develop, i.e., its potential connectivity, is much greater than the actual number of synapses, and structural plasticity allows the system to explore different wiring possibilities (Stepanyants et al., 2002; Fares and Stepanyants, 2009).

Here, we use an *adaptive*—sometimes also called *co-evolving*—brain network model, which has already been used by us to describe synaptic pruning in humans (Millán et al., 2018a,b), to analyze how the dynamical processes of adding and removing synapses during brain development can affect the ability of the network to store and optimally retrieve a given set of memories. Our system combines the auto-associative Amari-Hopfield neural network (Amari, 1972; Hopfield, 1982) with a preferential-attachment dynamics for the network evolution in a way that has been shown to accurately reproduce the observed variation of neuron connectivity data on human brains during infancy (Johnson et al., 2010; Millán et al., 2018b). As empirically observed—see (Holtmaat and Svoboda, 2009) and references therein—this model assumes that the probabilities of growth and death of synapses depend on both the mean connectivity in the system and the neural activity. Previous studies have analyzed the effect of thermal noise in the system and its emergent behavior, and they have shown that the coupling between neuronal activity and connectivity creates a feedback loop between form and function since the system activity influences its topology and, in turn, it is affected by the network structure through the synaptic currents the neurons receive (Millán et al., 2018a). As a matter of fact, depending on parameters, this system is then able to produce heterogeneous networks with the presence of hubs, similar to the ones observed in actual neural systems (Van Den Heuvel and Sporns, 2011; Crossley et al., 2014; Oh et al., 2014; Stafford et al., 2014), with high memory retrieval and noise tolerance. Another recent work has also studied the effect of a transient period of high connectivity before synaptic pruning begins (Millán et al., 2018b), as observed in mammal brains (Huttenlocher and Dabholkar, 1997; Navlakha et al., 2015), demonstrating that it has beneficial effects for memory recovery and the emergence of an organized stationary state in the system.

Here we develop on the effect that synaptic (or quenched) disorder resulting from the interference among many patterns of activity—stored by *Hebbian* learning on the synaptic weights—has on the emergent behavior of the system. We show that, as a consequence of the interplay between structural (i.e., pruning), thermal and quenched disorder, oscillations can emerge in the activity of the model which imply visiting different memorized patterns, an emergent behavior that had not been reported before in this model. This intriguing behavior is precisely due to long-term synaptic mechanisms associated with the network evolution dynamics, and not to short-term synaptic processes, such as synaptic depression and facilitation (Pantic et al., 2002; Marro et al., 2007; Torres et al., 2007, 2008; Torres and Marro, 2015) or spike frequency adaptation (Knoblauch and Palm, 2002; Ha and Cheong, 2017), which are not present in our model. These have already been described to induce oscillations among stored patterns of network activity, however the biophysical mechanisms behind them are different from the topological rewiring process considered here, and in particular they act on shorter time-scales—on the order of *ms* as opposed to the time scale of hours or days in which synaptic rewiring operates. It would be straightforward to extend the present study by adding short-term mechanisms, and we hypothesize that the interplay between different neuron and synaptic processes during learning and brain evolution could give rise to other types of oscillatory phenomena associated with non-equilibrium phases not yet reported, a fact that we glimpse could have strong computational implications.

## 2. Model and Methods

Our system consists in a time-dependent, symmetric, undirected, *N*-node complex network (Boccaletti et al., 2006) of neurons, defined at time *t* by the adjacency matrix *e*(*t*), with elements *e*_*ij*_(*t*) = {0, 1}, in which each node represents a neuron and each edge [*e*_*ij*_(*t*) = 1] stands for a synapse. The *degree* of node *i* at time *t* is defined as

(1) $$\begin{array}{l} k_{i}\left(t\right)=\overset{N}{\underset{j=1}{\sum }}e_{ij}\left(t\right) \end{array}$$

and the *mean degree* of the network is

(2) $$\begin{array}{l} \kappa \left(t\right)=\frac{1}{N}\overset{N}{\underset{i=1}{\sum }}k_{i}\left(t\right). \end{array}$$

Following a familiar (Amari-Hopfield) prescription (Amari, 1972; Amit, 1989), each neuron *i* is modeled as a stochastic binary unit, *s*_*i*_(*t*) = {0, 1} (representing respectively a silent and a firing neuron), whose state evolves in time according to the probabilistic dynamics

(3) $$\begin{array}{l} P\left[s_{i}\left(t+1\right)=1\right]=\frac{1}{2}\left\{1+\tanh\left[T^{-1}\left(h_{i}\left(t\right)-\theta_{i}\left(t\right)\right)\right]\right\}, \end{array}$$

where

(4) $$\begin{array}{l} h_{i}\left(t\right)=\overset{N}{\underset{j=1}{\sum }}w_{ij}e_{ij}\left(t\right)s_{j}\left(t\right) \end{array}$$

is the *local field* at neuron *i* quantifying the incoming input from neighbor neurons and

(5) $$\begin{array}{l} \theta_{i}\left(t\right)=\frac{1}{2}\overset{N}{\underset{j=1}{\sum }}w_{ij}e_{ij}\left(t\right) \end{array}$$

is the neuron's *threshold* for firing. This definition of the threshold is typically considered, in the case of static networks, when the more biologically plausible {0, 1} code is used instead of the canonical {±1} one, since it allows one to recover the phase diagram of the canonical, fully connected Amari-Hopfield model (Amit, 1989). Therefore, we maintain it when extending the model to a time dependent topology, and it naturally leads to a *dynamic threshold*. This is not a strong assumption since dynamic or adaptive thresholds have been widely described in several neural systems. For instance, they have been shown to create a nontrivial motion between the attractors of the system (Horn and Usher, 1989; Itskov et al., 2011) and to have a major role in stochastic resonance (Mejias and Torres, 2011) and in the functioning of sensory systems (Fricker et al., 1999; Azouz and Gray, 2000, 2003; Cardin et al., 2008; Kobayashi et al., 2009). Mechanisms of threshold adaptation have been found to help to avoid saturating activity during developmental changes (Turrigiano et al., 1998), and to be related to homeostatic regulation mechanisms observed in cortical neurons (Abbott and LeMasson, 1993; Turrigiano et al., 1998), and to the emergence of self-organized criticality in neural systems (Uhlig et al., 2013; Hobbiss et al., 2018). In our context, θ_*i*_(*t*) depends only on the existing synapses, which can be seen as a means of homeostasis since the response of a neuron is regulated by the number and strength of its synaptic contacts, thus avoiding silencing low-degree neurons and saturation of hubs. Furthermore, in our model the term *e*_*ij*_*w*_*ij*_ in Equation (5) characterizes the intensity of the synaptic transmission between neurons *i* and *j*, so that the threshold dynamics depends indirectly on the neural activity.

On the other hand, the noise parameter or *temperature T* (*T* > 0) sets the level of stochasticity on the activity of the neurons, so that if *T* = 0 the evolution of the system is deterministic and the state of a neuron at time *t* is completely determined by the states of its neighbors at time *t* − 1. For *T* > 0, however, the evolution is stochastic and, as *T* is increased, the thermal noise has a stronger effect. The strength of each synapse, or its *synaptic weight*, *w*_*ij*_, is a real variable defined by means of a set of *P* binary patterns of neural activity, $\xi_{i}^{\mu }\in \left\{0,1\right\}$, μ = 1, …, *P*, according to the Hebbian learning prescription (Amit, 1989),

(6) $$\begin{array}{l} \begin{array}{c} w_{ij}={\left[\kappa_{0}a_{0}\left(1-a_{0}\right)\right]}^{-1}\overset{P}{\underset{\mu =1}{\sum }}\left(\xi_{i}^{\mu }-a_{0}\right)\left(\xi_{j}^{\mu }-a_{0}\right),\;i\neq j\\ w_{ii}=0, \end{array} \end{array}$$

where κ_0_ = κ(*t* = 0) and *a*_0_ is the mean activation of the patterns, i.e., $a_{0}={\left(NP\right)}^{-1}\overset{P}{\underset{\mu =1}{\sum }}\overset{N}{\underset{i=1}{\sum }}\xi_{i}^{\mu }$. This definition of the synaptic weights makes the patterns $\xi_{i}^{\mu }$ attractors of the activity dynamics of the system, and therefore it constitutes the final step of a process of “learning” or “storing” of a set of activity patterns by the system in the synaptic weights. Notice also that *w*_*ij*_ = *w*_*ji*_ by construction so that the network is symmetric, in the spirit of previous studies (Sompolinsky and Kanter, 1986). This is for simplicity and also as a reference to compare with the canonical Amari-Hopfield model (Amari, 1972; Hopfield, 1982).

The *overlap* of the network state with each of these patterns determines the global state of the system, and it is defined as

(7) $$\begin{array}{l} m^{\mu }\left(t\right)={\left[Na_{0}\left(1-a_{0}\right)\right]}^{-1}\overset{N}{\underset{i=1}{\sum }}\left(\xi_{i}^{\mu }-a_{0}\right)s_{i}. \end{array}$$

It follows from this definition that −1 ≤ *m*^μ^(*t*) ≤ 1. We say that the system is in a *memory state* or, equivalently, that it has *retrieved* pattern μ, if *m*^μ^ > 2/3 and *m*^ν^ → 0 ∀ν ≠ μ. This indicates that the activity state of the network strongly resembles that of pattern μ. In the case of a non-trivial topology, it is also of interest the degree dependent overlap, *m*^μ^(*k, t*), defined as

(8) $$\begin{array}{l} m^{\mu }\left(k,t\right)={\left[Np\left(k,t\right)a_{0}\left(1-a_{0}\right)\right]}^{-1}\overset{N}{\underset{i=1}{\sum }}\left(\xi_{i}^{\mu }-a_{0}\right)s_{i}\delta_{k,k_{i}} \end{array}$$

where *p*(*k, t*) is the degree distribution of the network, which indicates the probability that a node has degree *k* at a certain time *t* (*p*(*k, t*) ≥ 0 ∀*k, t*, $\overset{N}{\underset{k=1}{\sum }}p\left(k,t\right)=1\;\forall t$). Therefore, $m^{\mu }\left(t\right)=\overset{N}{\underset{k=1}{\sum }}p\left(k,t\right)m^{\mu }\left(k,t\right)$. Notice also that if the patterns of activity are not homogeneously distributed through the neurons, *m*^μ^(*k, t*) is not bounded by ±1.

The “canonical” setting of the Amari-Hopfield model, in the case of a fully connected network and random orthogonal patterns, exhibits three characteristic phases. In the absence of thermal noise, *T* = 0, the patterns $\xi_{i}^{\mu }$ are stable attractors of the dynamics of the system for *P* < *P*_*c*_ = 0.138*N*, and the system is in what is called the *memory phase. P*_*c*_ defines the *maximum storage capacity* of the network (Amit, 1989), that is, the maximum quantity of information—or number of patterns—that can be stored and effectively retrieved from the network. This phase is (mathematically) equivalent to the *ferromagnetic* or *ordered phase* of interacting spin networks (as in the Ising model). The storage of a large number of different patterns in the network gives rise to *quenched noise* as a consequence of the interference between them in *w*_*ij*_, which can destabilize such memory phase. Therefore, if *P* is further increased above *P*_*c*_ there is a discontinuous phase transition to a *spin-glass (SG) phase*, in which there appear metastable states which are combinations of the stored patterns (therefore called *mixed states*) that trap the dynamics of the system. Similarly, in the case of *P* = 1 when *T* > *T*_*c*_ = 1, there is a continuous phase transition from the memory phase to a noisy or *paramagnetic phase* (also called *disordered phase*) in which there are no stable attractors, and the dynamics of the system is driven by noise (Amit, 1989). In the more general case in which both *T* > 0 and *P* > 1, the location of the phase transitions depends both on *T* and *P*. The emergent behavior of the Amari-Hopfield model has also been studied on non-trivial network topologies, such as scale-free and small-world networks (Torres et al., 2004; Boccaletti et al., 2006; Oshima and Odagaki, 2007). Such systems have been shown to present the same phases as the canonical fully connected model, with transition lines that depend on the topology. In particular, it has been reported that, for heterogeneous networks and a single stored pattern, the overlap reduces for *T* < *T*_*c*_, so that memory is recovered but with more errors than in a fully connected network. However, the critical temperature diverges, *T*_*c*_ → ∞ as *N* → ∞, due to the presence of hubs that retain pattern information. Therefore, the memory phase expands to much higher values of thermal noise. On the other hand, the capacity of the network is known to decrease as the mean connectivity of the network decreases (Torres et al., 2004).

We here consider an evolving network whose structure, contrary to the canonical model above, changes constantly in time subjected to the pruning dynamics, as we shall describe. Moreover, we consider a highly sparse network, with values of κ/*N* ∈ [10^−3^, 10^−2^], which can be homogeneous (i.e., every node having roughly the same connectivity degree), or heterogeneous, with the formation of hubs. Both sparseness and heterogeneity damage severely the memory retrieval ability of the neural network that, for such cases, diminishes fast with *P* compared with the case of highly connected and homogeneous neural networks (Stauffer et al., 2003; Castillo et al., 2004; Morelli et al., 2004; Torres et al., 2004; Oshima and Odagaki, 2007; Akam and Kullmann, 2014) However, there is experimental evidence that the configurations of neural activity related to particular memories in the animal brain involve many more silent neurons, $\xi_{i}^{\mu }=0$, than active ones, $\xi_{i}^{\mu }=1$ (Chklovskii et al., 2004; Akam and Kullmann, 2014). Notice that in this case there is a positive correlation between different patterns due to the sparseness, since *a*_0_ ≠ 0.5, which is also known to improve the storage capacity of a neural network (Knoblauch et al., 2014; Knoblauch and Sommer, 2016), and in particular that of heterogeneous and sparse neural networks (Morelli et al., 2004). Consequently, we consider here this kind of activity patterns, and we further define them as non-overlapping regions of active neurons, each consisting of *N*/*P* neurons, so that they cover the whole network (and therefore the mean activity of the patterns is $a_{0}=P^{-1}$). This corresponds to a particular definition of sparse or biased patterns, which in other works have been considered to be randomly distributed with a given *a*_0_ (Knoblauch et al., 2014; Knoblauch and Sommer, 2016), what allows for a good visualization of the activity of the network by means of the raster plots.

Moreover, this scheme allows us to define another measure of the overlap between the state of the system and the memorized patterns, considering only the corresponding active neurons as

(9) $$\begin{array}{l} m_{1}^{\mu }\left(t\right)\equiv \frac{1}{N}\overset{N}{\underset{i=1}{\sum }}s_{i}\left(t\right)\xi_{i}^{\mu }, \end{array}$$

with $m_{1}^{\mu }\in \left[0,1\right]$. If is also of interest its binearized extension, $m_{B}^{\mu }$, defined as $m_{B}^{\mu }\left(t\right)\equiv 1$ if $m_{1}^{\mu }\left(t\right)>m_{th}$ and 0 otherwise, so that $m_{B}(t)=\left(m_{B}^{1}(t),m_{B}^{2}(t),...,m_{B}^{P}(t)\right)$ indicates, in a binary code, which combination of patterns is recovered at time *t*. Equivalently, the decimal variable *d*_*s*_ can be defined,

(10) $$\begin{array}{l} d_{s}\left(t\right)\equiv \overset{P}{\underset{\mu =1}{\sum }}2^{\mu -1}m_{B}^{\mu }\left(t\right), \end{array}$$

which one can interpret as a one-dimensional variable indicating the global memory state of the system.

Interestingly, the activity patterns defined here are such that when a number *P*_*r*_ of them are recovered at the same time, in a SG-like state, the maximum overlap that they can have is less than one. In order to see this, one can decompose Equation (7) in *P* sums, each over the neurons corresponding to the region associated with each of the activity patterns, as

(11) $$\begin{array}{l} m^{\mu }={\left[Na_{0}\left(1-a_{0}\right)\right]}^{-1}\overset{P}{\underset{\nu =1}{\sum }}\overset{N}{\underset{i=1}{\sum }}\left(\xi_{N\nu -N+i}^{\mu }-a_{0}\right)s_{N\nu -N+i}, \end{array}$$

where $N=a_{0}N=N/P$ is the size of each region and the time dependency has been dropped for clarity. Here, the first sum is over the *P* patterns stored in the network, whereas the second one goes over the N neurons in the region associated with each pattern. If the pattern μ is recovered together with other *P*_*r*_−1 patterns, then the sum over ν can be split in three terms: the region associated with the pattern μ, the ones corresponding to the other retrieved patterns, and finally those of the non-retrieved patterns (which do not contribute to the sum). Therefore, the overlap corresponding to this pattern is $m^{\mu }={\left(1-P^{-1}\right)}^{-1}\left[1-P^{-1}-\left(P_{r}-1\right)P^{-1}\right]$. This yields

(12) $$\begin{array}{l} m^{\mu }=1-\left(P_{r}-1\right){\left(P-1\right)}^{-1}\leq 1, \end{array}$$

which only meets the equality in the case *P*_*r*_ = 1, that is, if only the pattern μ is retrieved.

The network structure changes in time following a preferential attachment process. This is characterized by the probability each node *i* has to gain or lose an edge at each time *t* – namely,

(13) $$\begin{array}{l} \begin{array}{c} P_{i}^{g}=u\left(\kappa \right)\pi \left(I_{i}\right),\\ P_{i}^{l}=d\left(\kappa \right)\eta \left(I_{i}\right), \end{array} \end{array}$$

where *I*_*i*_ = |*h*_*i*_ − θ_*i*_| is the scaled input that each neuron receives as a consequence of the coupling with its neighbors, a sort of recurrent current in the network, and the time dependence has been dropped for clarity. Here, *u* and *d* account for global factors that affect synaptic growth and death, such as the diffusion of different molecules through large areas of tissue, for which the mean degree κ is taken as a proxy. The second terms π and η introduce a dependence on the pre-synaptic activity of the nodes, closing the activity-topology coupling. This creates a feedback loop between the evolution of the structure of the network (form), mediated by the local currents, and the neural activity on the network (function).

Taking the local probabilities to be normalized over the network, the number of edges that are added and removed at each time *t* depends only on the global probabilities *u*(κ(*t*)) and *d*(κ(*t*)). In this way, they determine the temporal evolution of the mean connectivity κ(*t*), whereas the local probabilities π(*I*_*i*_) and η(*I*_*i*_) characterize the second order statistics of the network structure, such as the variance of the degree distribution or the degree-degree correlations, as we show below (see also Millán et al., 2018a). These definitions allow us to simulate the dynamics of the system via a Monte Carlo method (in particular, we make use here of the BKL algorithm Bortz et al., 1975) as follows. First, the number of edges to be created and destroyed at time *t* is sorted according to the global probabilities *u*(κ(*t*)) and *d*(κ(*t*)). Then, we select as many nodes as indicated with these draws, independently of each other, according to π(*I*_*i*_) and η(*I*_*i*_). This process is done in a serial manner, and the same node can be selected more than once. Notice that for each node *i* that gains or looses an edge *e*_*ij*_, the degree of the second node *j* to which that edge links also changes accordingly. Therefore, there are in fact two paths that can lead to the change of a node's degree: either through the primary process with probability π(*I*_*i*_) for a gain (or η(*I*_*i*_) for a loss), or when it is randomly connected to (or disconnected from) an already chosen node. Therefore, the effective values of the second factors in Equation (13) are

(14) $$\begin{array}{l} \begin{array}{c} {\tilde{\pi }}_{i}=\frac{1}{2}\left[\pi \left(I_{i}\right)+\frac{1}{N}\right],\\ {\tilde{\eta }}_{i}=\frac{1}{2}\left[\eta \left(I_{i}\right)+\frac{k_{i}}{\kappa N}\right], \end{array} \end{array}$$

where the 1/2 factor is included to assure normalization. Following our previous work, we consider

(15) $$\begin{array}{l} \begin{array}{c} {\tilde{\pi }}_{i}=\frac{I_{i}^{\alpha }}{\langle I^{\alpha }\rangle N},\\ {\tilde{\eta }}_{i}=\frac{I_{i}}{\langle I\rangle N}, \end{array} \end{array}$$

which are normalized over the network, $\overset{N}{\underset{i=1}{\sum }}{\tilde{\pi }}_{i}=\overset{N}{\underset{i=1}{\sum }}{\tilde{\eta }}_{i}=1$. The power-law relation in ${\tilde{\pi }}_{i}$ allows us to explore both sub- and super-linear responses by just modifying a single parameter, namely α. The probability ${\tilde{\eta }}_{i}$, on the other hand, is fixed in a linear response, which corresponds to edges being chosen at random for removal, which can be seen as a first order approximation to the pruning dynamics (Millán et al., 2018a). Therefore, α is the control parameter for the pruning dynamics. If α < 1, high degree nodes are more likely to lose edges than to gain new ones, thus creating a homogeneous network structure. On the other hand, if α > 1, high degree nodes are more likely to continue to gain edges than to lose them, which gives rise to a highly heterogeneous, bimodal structure. Finally, the case α = 1 corresponds to the critical case in which networks develop a scale-free topology as shown in previous works (Johnson et al., 2010) that reproduces the scaling behavior observed in the long-range connections of the human brain (Gastner and Ódor, 2016) and in protein interaction networks (Albert, 2005), which decay as a power-law with exponent μ ≈ 2.5.

The local probabilities are then given by

(16) $$\begin{array}{l} \begin{array}{c} \pi \left(I_{i}\right)=\max\left\{2\frac{\overset{\alpha }{\underset{i}{I}}}{\langle I^{\alpha }\rangle N}-\frac{1}{N},0\right\},\\ \eta \left(I_{i},k_{i}\right)=\max\left\{2\frac{I_{i}}{\langle I\rangle N}-\frac{k_{i}}{\kappa N},0\right\}, \end{array} \end{array}$$

which hold that π(*I*_*i*_), η(*I*_*i*_, *k*_*i*_) > 0 ∀*i* and normalization, $\overset{N}{\underset{i=1}{\sum }}\pi \left(I_{i}\right)=\overset{N}{\underset{i=1}{\sum }}\eta \left(I_{i},k_{i}\right)=1$.

We impose further restrictions on the network. First of all, *e*_*ij*_ is a binary matrix, so that only one edge per pair of nodes is allowed and the strength of the connection between two neurons, resembling the number of multiple contacts between actual neurons (Fares and Stepanyants, 2009), is considered to be given by *w*_*ij*_. Moreover, we set the minimum degree of the network, *k*_*i*_ = 1, so that there cannot be any disconnected nodes, and we forbid self-connections, *e*_*ii*_ = 0 ∀*i*. The maximum degree a node can have is therefore *N*−1. We do not impose a hard bound on it as other works have done (Knoblauch et al., 2014; Knoblauch and Sommer, 2016). This would exclusively affect hubs, which only appear for α > 1, as discussed above (Johnson et al., 2010; Millán et al., 2018a), reducing their connectivity. This might affect the memory capabilities of the network in the limit *P* → ∞ but, since we do not work on this limit, we do not expect any changes on the qualitative behavior and main findings of our model.

Under this framework, the evolution of the mean degree is

(17) $$\begin{array}{l} \frac{d\kappa \left(t\right)}{dt}=2\left[u\left(\kappa \left(t\right)\right)-d\left(\kappa \left(t\right)\right)\right]. \end{array}$$

For a careful derivation of this equation, we direct the reader to Johnson et al. (2010). Intuitively, *u*(κ(*t*)) and *d*(κ(*t*)) set the number of edges that are created and destroyed at every time step, so that *u*(κ(*t*))−*d*(κ(*t*)) gives the net change in the number of edges. Since for each of these edges two nodes change their degree, there is a factor 2 in the variation of the mean degree. The simplest way to approximate the pruning dynamics is to consider an exponential decay of κ(*t*) from κ_0_ to κ_∞_, where κ_0_ = κ(*t* = 0) is the initial mean degree of the network and κ_∞_ = κ(*t* → ∞) the stationary mean degree after synaptic pruning has occurred, so that κ_0_ ≥ κ_∞_. This is achieved by defining

(18) $$\begin{array}{l} \begin{array}{c} u\left(\kappa \left(t\right)\right)=\max\left\{\frac{n}{N}\left(1-\frac{\kappa \left(t\right)}{2\kappa_{\infty }}\right),0\right\}\\ d\left(\kappa \left(t\right)\right)=\frac{n}{N}\frac{\kappa \left(t\right)}{2\kappa_{\infty }}, \end{array} \end{array}$$

where the parameter *n* sets the timescale for the pruning dynamics. Notice also that Equation (18) assures that *u*(κ) ≥ 0 ∀κ. By substituting these definitions into Equation (17), we obtain the time evolution of κ(*t*),

(19) $$\begin{array}{l} \kappa \left(t\right)=\kappa_{\infty }\left[1-\left(1-\kappa_{0}/\kappa_{\infty }\right)e^{-t/\tau_{p}}\right], \end{array}$$

where τ_*p*_ = *Nκ*_∞_/(2*n*). This set-up has been previously used to reproduce experimental data on the connectivity of the human pre-frontal cortex using values of κ_0_ ∈ (60, 80) and κ_∞_ ∈ (30, 50), depending on the region, and also of the mouse somato-sensory cortex, with κ_0_ = 3.10 and κ_∞_ = 1.64 (for the other parameters see Johnson et al., 2010; Millán et al., 2018a). The definitions in Equation (18) take into account that synaptic growth and death relay in some way on the concentrations of various molecules (that can have an important role in synaptogenesis, as axonal growth factors), which can diffuse through large areas of tissue and therefore cannot in general be considered local (Klintsova and Greenough, 1999), and here we consider κ(*t*) as a proxy for the amount of resources consumed by the existing synapses in the network. In an environment with a finite presence of nutrients, it is reasonable to think that there is a competition for the existing resources, and that neurons are sensitive to the amount of nutrients available to them, so that synapses are less likely to grow, and more likely to atrophy, when the connectivity is high, and viceversa, as assumed by Equation (18).

Finally, the network macroscopic state is described via the degree distribution *p*(*k, t*) and its *homogeneity*, defined as

(20) $$\begin{array}{l} g\left(t\right)=\exp\left(-\sigma^{2}\left(t\right)/\kappa^{2}\left(t\right)\right), \end{array}$$

where σ^2^(*t*) = 〈*k*^2^(*t*)〉 − κ^2^(*t*). For homogeneous networks, in which all nodes have similar degrees, σ(*t*) is small and the homogeneity approaches one, with the trivial case of *g* = 1 if *p*(*k, t*) = δ_*k*,_*k*__1__. For heterogeneous networks, on the other hand, there are big fluctuations in the degrees of the nodes and *g*(*t*) → 0.

The timescale for structure changes is set by the parameter *n*, whereas the time unit for activity changes, *h*_*s*_, is the number of Monte Carlo Steps (MCS) that the states of all neurons are updated according to the Amari-Hopfield dynamics between each structural network update. Our studies show a low dependence on these parameters in the cases of interest, so we only report here results for *h*_*s*_ = 10 MCS and *n* = 10. Measures on the stationary state of the system are carried out by temporal averages of the macroscopic variables, $\bar{f}=\Delta \overset{t_{0}+\Delta }{\underset{t=t_{0}}{\sum }}f\left(t\right)$.

A recent work (Millán et al., 2018a) showed that, within this framework, three phases emerge: a homogeneous memory phase when both α and *T* are low (*T*, α < 1), in which the network is capable of memory retrieval and the topology dynamics keeps a homogeneous configuration; a heterogeneous memory phase for high α (α > 1) in which the dynamics leads to bimodal networks (with the appearance of hubs or highly connected nodes); and a homogeneous noisy phase for high noise *T*. However, as we will depict in the next section, the combination of thermal noise together with the introduction of a larger number of patterns of activity—which induces interference among them—induces other non-reported non-equilibrium phases characterized by the emergence of complex oscillations among the activity associated with the stored patterns.

## 3. Results

Previous preliminary analysis of the storage capacity of developing brains under the present framework revealed that the capacity of the network can be greatly improved if a feedback loop between structure and function is considered (Millán et al., 2018a). This is because the interplay between form and function gives rise to a topological structure that enhances the stability of the memory attractors which are recovered during the evolution of the system. In order to explore this interesting picture under other conditions, here we analyze in detail the phase diagram of the system with respect to four relevant parameters in the model, namely, α, κ_∞_, *T*, and *P*. The first two characterize the network structure dynamics, whereas the temperature, *T*, and the number of stored patterns, *P*, account respectively for thermal and quenched disorder. As already said, the latter is a consequence of the interference among many stored patterns, and it can affect the recall process. Other parameters, such as the initial connectivity κ_0_ or the speed of the pruning, *n*, where shown to have little or no effect on the dynamics (Millán et al., 2018a,b).

### 3.1. Steady State Solutions for *T* = 0

We first analyze the behavior of the system at *T* = 0, that is, in absence of thermal fluctuations that can affect the stability of the fixed point solutions of the system dynamics. As stated above, there are, however, other sources of noise in our system which can have a prominent influence in its behavior. One is the interference among stored patterns, which can significantly reduce the memory retrieval ability of the system (Amit, 1989). Another is the pruning dynamics that adds a second source of noise; this is an intrinsic, structural noise that emerges due to the stochastic adding and removal of synapses associated with the network dynamics during brain development, and which can dynamically affect the performance of the system during memory acquisition and consolidation.

**Figure 1:**
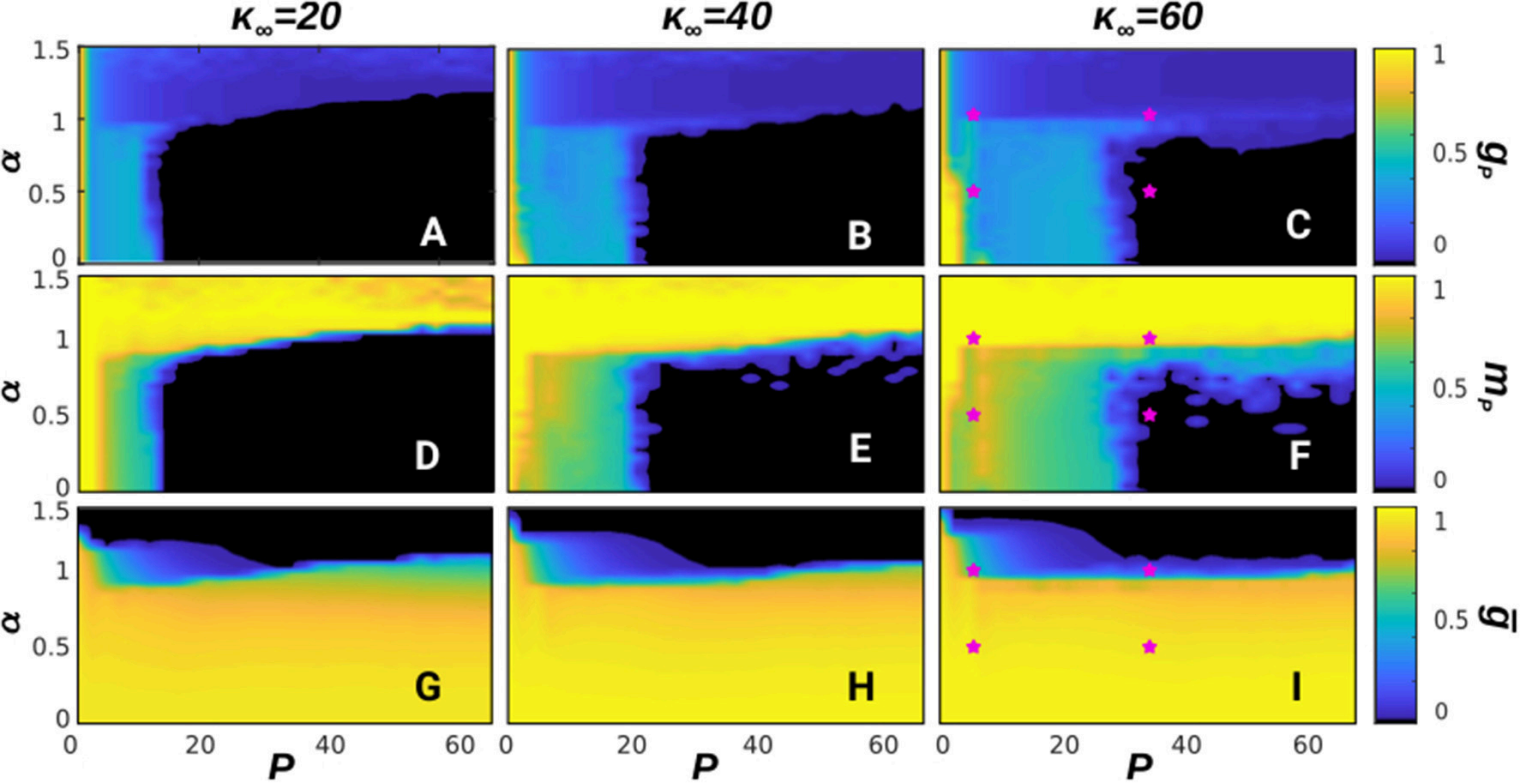
Phase diagrams depicting the steady-state of the system with respect to *P* and α at *T* = 0. The first row of panels **(A–C)** shows the fraction of patterns, *g*_*P*_, retrieved after a given transient; the second one **(D–F)** shows the average overlap with the recovered patterns, *m*_*P*_; and finally the third one **(G–I)** shows the stationary homogeneity *ḡ*. Each column is for a different value of κ_∞_, with κ_∞_ = 20, 40, and 60, respectively from left to right. The network size was set here to *N* = 1, 600 and each point has been averaged over 10 realizations of the system. In this figure a memory phase appears as a blue region in the diagram of *g*_*p*_ and a high value of *m*_*p*_, indicated by a yellow color. A SG phase appears as a blue region in *g*_*p*_ and a lower value of *m*_*p*_, indicated by a green color, whereas a noisy phase appears as black in *g*_*p*_ and *m*_*p*_. Similarly, homogeneous structures take place for high values of *ḡ*, indicated by a yellow region in the corresponding diagram, whereas heterogeneous structures are for low values of *ḡ*, indicated by a black region. Finally, the pink stars in diagrams **(C,F,I)** indicate the (*P*, α) points corresponding to the time series shown in Figure 2.

In Figure 1 we show the corresponding phase diagrams of the system (depicting different phases or kinds of behavior) for different values of κ_∞_ = 20, 40, and 60, respectively from left to right. These depict some non-equilibrium phases associated with different computational abilities during memory recall. The top panels show, in the steady state, the ratio of patterns that can be retrieved with high overlap (*m*^μ^ ≥ 0.66), namely *g*_*P*_ ≡ *P*_*r*_/*P* (where *P*_*r*_ is the number of retrieved patterns), as a function of α and *P*. A value *g*_*P*_ = 1/*P* indicates a pure memory state, whereas larger values correspond to mixtures and SG-like states (Amit, 1989), and *g*_*P*_ = 0 corresponds to the noisy or non-memory state. Meanwhile, the middle panels show the mean overlap of the recovered patterns during memory recall, namely *m*_*P*_ and, finally, the bottom panels show the stationary mean homogeneity, *ḡ*.

**Figure 2:**
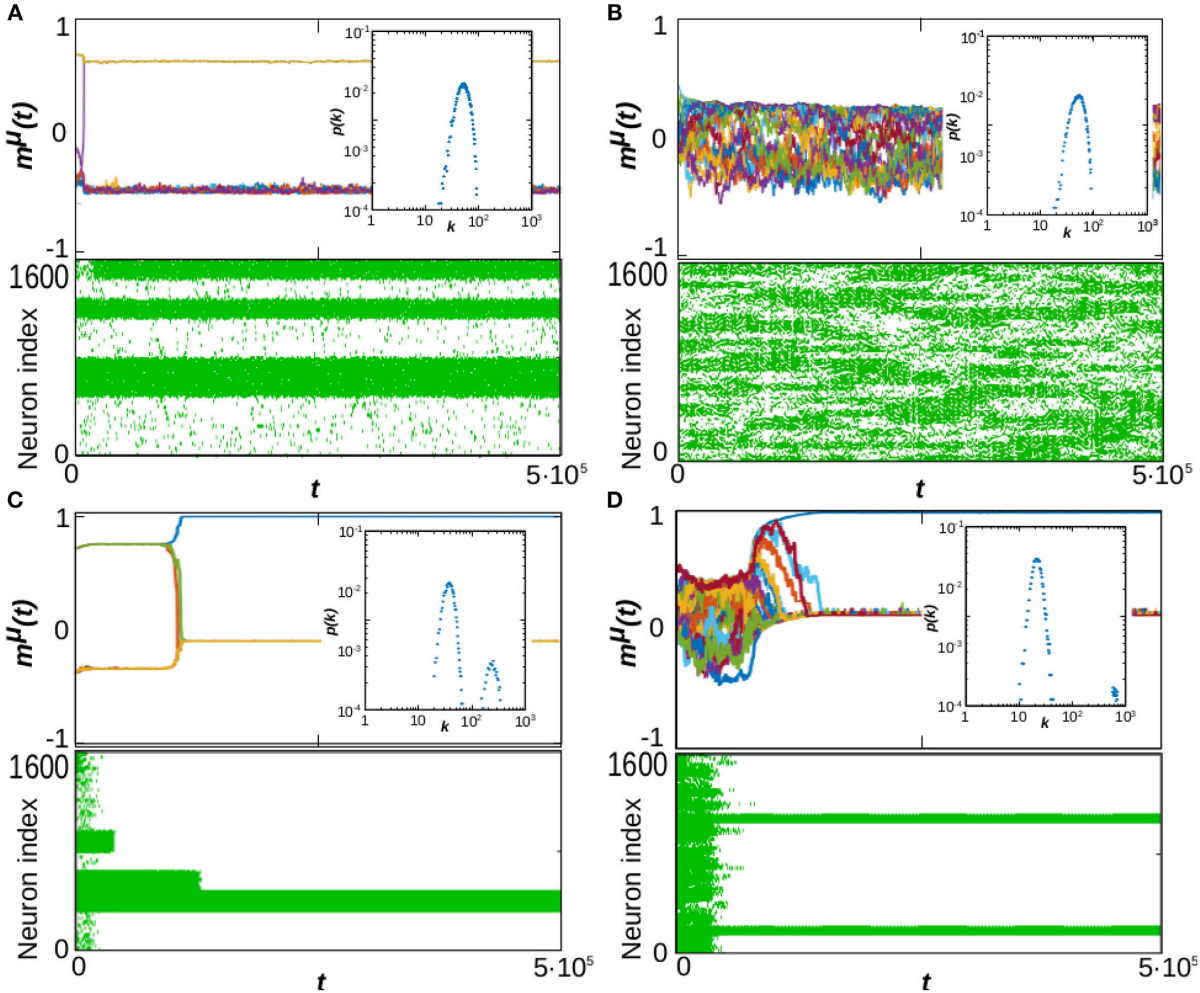
Time evolution of the system at *T* = 0 and κ_∞_ = 60 in four typical cases corresponding to different values of α and *P* as marked with pink stars in the phase diagrams of Figure 1. Each composite panel illustrates the overlap time series, *m*^μ^(*t*), (top graph), raster plots of neuron activity (bottom graph) and the steady-state degree distribution of the network (inset), computed at *t* = 10^6^ Monte Carlo Steps and averaged over 10 realizations of the system. The panels correspond respectively to α = 0.5 and *P* = 10 **(A)**, α = 0.5 and *P* = 30 **(B)**, α = 1.5 and *P* = 10 **(C)**, and α = 1.5 and *P* = 30 **(D)**. In all presented simulations we set *N* = 1, 600.

These diagrams show up different types of dynamical behavior. In order to illustrate the characteristics of each one, in Figure 2 we depict the time series *m*^μ^(*t*) (top graph of each panel), raster plots showing the whole activity of the system (bottom graph of each panel) and the steady-state degree distribution (inset of each panel) for some particular values of α and *P* corresponding to different characteristic behaviors in the phase diagrams in Figure 1. For a given stationary connectivity (e.g., κ_∞_ = 60, Figures 1C,F,I) we find that, for *P* = 1, the system is able to retain memory for almost every value of α, as it can be seen by the yellow region at *P* = 1 in Figure 1F, which indicates an overlap equal to 1. For small *P* and small α, SG-like states, or mixture states (in which some of the memories are partially retrieved at the same time), start to emerge as it is illustrated in Figure 2A, which corresponds to the point α = 0.5, *P* = 10. As a consequence, both *g*_*P*_ and *m*_*P*_ take intermediate values; the former since only a finite number of patterns is retrieved, *g*_*p*_ < 1 (light blue region of the diagram in Figure 1C), the later because these are retrieved at the same time, and therefore the overlap is reduced, *m*_*p*_ < 1 (green and light-blue region of the diagram in Figure 1F). In general, however, the observed SG-like states present high values of the overlap with all the recovered patterns due to the high correlation between memories we have considered in this work. Moreover, in this region the network structure is homogeneous since α < 1, so that *ḡ* approaches 1 and the degree distribution resembles a Poisson distribution (see Figure 1I and the inset of Figure 2A). In these conditions, when *P* is increased the memories lose stability until there is a transition from the SG-like state to the noisy one, where the network structure remains homogeneous, as shown in Figure 2B for the point α = 0.5 and *P* = 30. This is indicated by *g*_*P*_ → 0 (black region in Figure 1C), *m*_*P*_ → 0 (black region in Figure 1F), and *ḡ* → 1 (yellow region on the bottom-right side of Figure 1I).

On the other hand, for high α (α > 1), just one (or very few) pattern is retrieved, with *m*_*P*_ ≈ 1, and the network structure becomes heterogeneous since α > 1 (see inset of Figure 2C). As a consequence, *g*_*P*_ → 1/*P*, (dark-blue region in Figure 1C), *m*_*P*_ approaches 1 (yellow region in Figure 1F), and *ḡ* → 0 (black region in Figure 1I). Memory is achieved due to heterogeneity and the presence of hubs, which can maintain the information content of the retrieved pattern even in the presence of the strong noise induced by the interference with other stored patterns and the dynamic changes of the network structure. Therefore, when *P* is increased the recovered patterns remain stable, so that *m*_*P*_ remains close to 1 (Figure 1F) and *g*_*P*_ decreases as 1/*P* since only one pattern is retrieved (Figure 1C, see also the inset of Figure 2D, showing the appearance of hubs, and Figure 1I, indicating *ḡ* → 0).

The stationary mean connectivity of the network, κ_∞_, also affects the behavior of the system, as it determines location of the phase transition from the SG phase to the noisy one for α < 1. As the diagrams in Figure 1 show, larger values of κ_∞_ increase the tolerance of the system to quenched disorder, so that a bigger number of patterns can be stored. This is in line with the known result that the information is stored in the synaptic weights, and therefore increasing the number of synapses also increases the amount of information that the system can store (Amit, 1989).

Notice also that the qualitative state of the system is approximately independent of *P* for *P* > 20, as shown in Figure 1 where one can see that *g*_*p*_, *m*_*p*_ and *ḡ* remain essentially constant as *P* is increased with constant α above *P* = 20, in agreement with previous studies (Millán et al., 2018a). Therefore, in the following we restrict our analysis to the most interesting region *P* < 20 and do not analyze the large storage limit of the system (Knoblauch et al., 2014; Knoblauch and Sommer, 2016). This is because our interest here is in characterizing the dynamic behavior arising as a consequence of the interplay between structure and function under the presence of thermal and quenched noise, rather than its storage capacity. Similarly, the inclusion of a hard bound on the maximum degree of the nodes would primarily affect the degrees of the hubs of bimodal networks. However, these typically form a highly connected core in the network, so the average path length between nodes would not increase heavily. Therefore, we expect that this bound would not have an important effect in the regime *P* ≪ *N* in which we set the system here.

In summary, for *T* = 0, that is, when there are only two sources of noise in the system (structural and quenched disorder), the stationary state for a given *P* depends strongly on the network structure, determined by α. As so, for α > 1, the network develops heterogeneous structures in which hubs arise. These are very densely connected with the rest of the network, and can maintain information about the memories even when *P* is very high. For α < 1, on the other hand, the network is always homogeneous, with every node having similar, low degree, and a SG-like phase soon arises, which is then suddenly lost as the quenched disorder becomes too strong and finally the system falls into the noisy state.

### 3.2. Behavior of the System for *T* > 0

**Figure 3:**
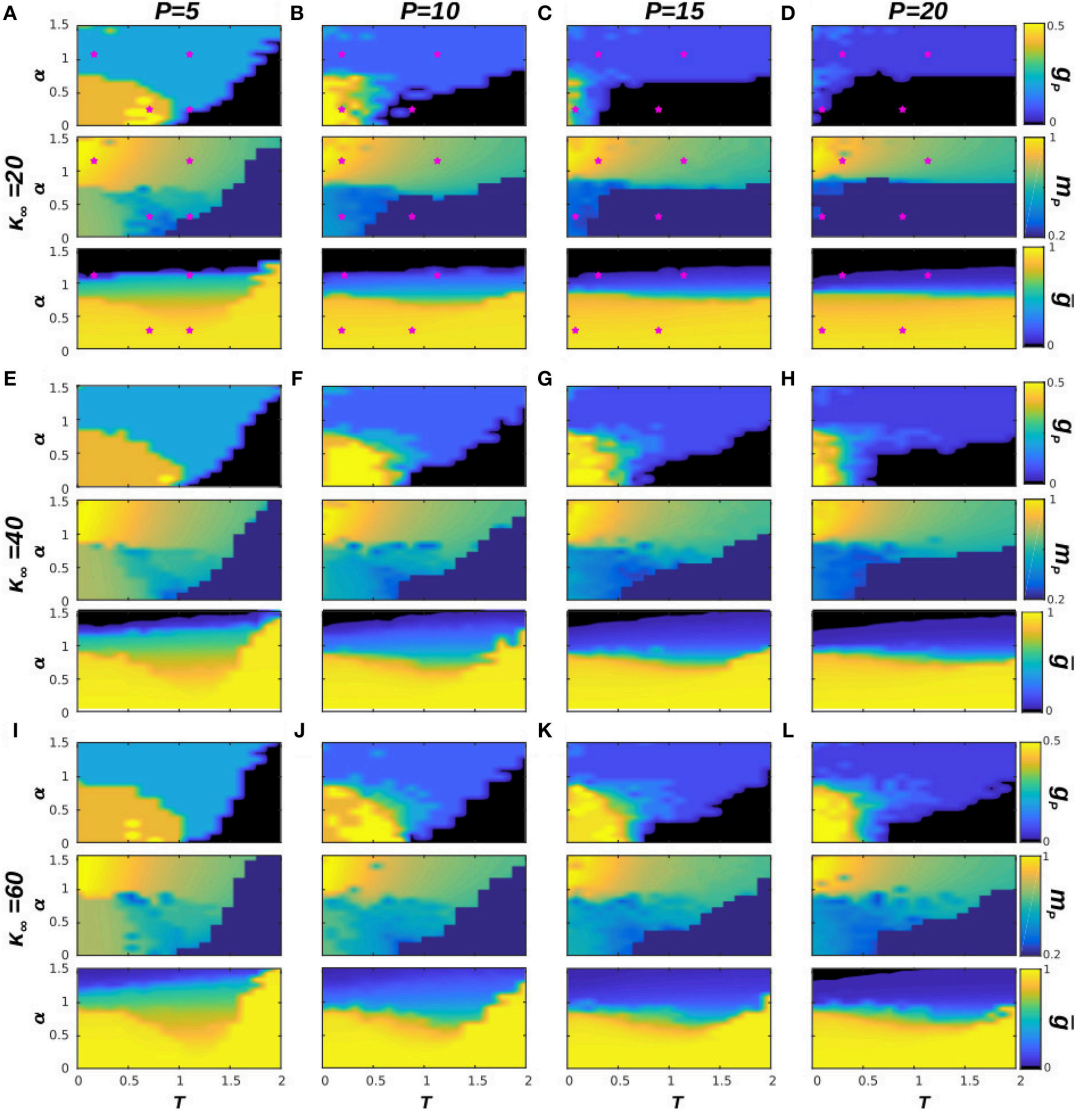
Phase diagrams of the system with respect to α and *T* for four different values of *P*, in particular for *P* = 5, 10, 15, and 20, respectively from left to right, and for three values of κ_∞_ = 20, 40, 60, respectively from top to bottom **(A–L)**. In each panel we show three diagrams: *g*_*p*_, *m*_*p*_, and *ḡ*, as indicated in the label of the color bar. Pink stars in **(A–D)** indicate the (*T*, α) point of the corresponding time series in Figure 4. Results are for *N* = 1, 600 and have been averaged over 5 realizations of the system dynamics. In this figure a memory phase appears as a blue region in the diagram of *g*_*p*_ and a high value of *m*_*p*_, indicated by a yellow or green color. A SG phase appears as an orange region in *g*_*p*_ and a lower value of *m*_*p*_, indicated by a green or blue color, whereas a noisy phase appears as black in *g*_*p*_ and dark-blue in *m*_*p*_. Finally, the oscillatory phase appears for high values of *g*_*p*_, (light yellow regions in the corresponding diagrams) and relatively low values of *m*_*p*_ (associated blue regions of the corresponding diagrams). Similarly, homogeneous structures take place for high values of *ḡ*, indicated by a yellow region in the corresponding diagram, whereas heterogeneous structures are for low values of *ḡ*, indicated by a black or dark blue region.

**Figure 4:**
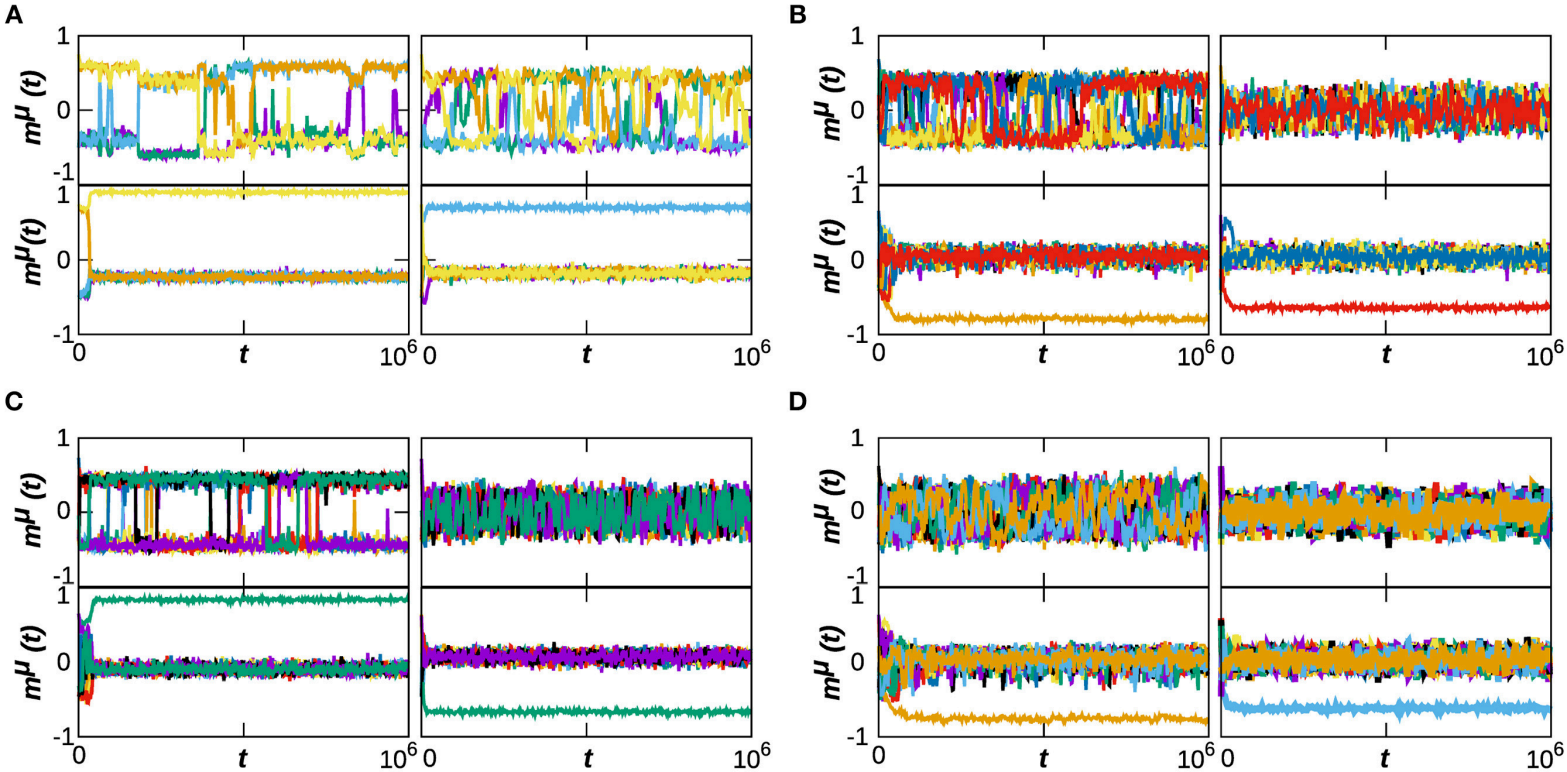
Time series of the overlap *m*^μ^(*t*) for some representative cases of the system dynamical behavior, corresponding to κ_∞_ = 20 and to *P* = 5, 10, 15, and 20, respectively from **(A–D)**. In each composite panel, we illustrate the behavior of the system on for 4 points of the (*T*, α) space, as indicated by pink starts in the corresponding phase diagrams of Figure 2. Namely, **(A)** is for the points (0.7, 0.3), (0.9, 0.3), (0.3, 1.1), (1.1, 1.1); **(B)** is for (0.3, 0.3), (0.9, 0.3), (0.3, 1.1), (1.1, 1.1); and finally **(C,D)** are for the points (0.1, 0.3), (0.9, 0.3), (0.3, 1.1), (1.1, 1.1). We have selected slightly different points for each *P* so as to show an example of the oscillatory behavior in each case, and the region of its appearance depends on *P*. Results are for *N* = 1, 600.

Our previous analysis has determined the phase diagram of the system at *T* = 0, which characterizes the effect of the dynamical topological structure on the memory capabilities of the system. In this section, we consider the effect of thermal noise in our system's emergent behavior. In order to do so, we analyze in Figure 3 the phase diagrams of the system with respect to α and *T*, for some representative numbers of stored patterns, namely *P* = 5, 10, 15, and 20 (each column of the figure corresponds to a different *P*), and for three values of κ_∞_ = 20, 40, and 60, as before. The selected values of *P* correspond to the left region of diagrams in Figure 1, where the phase transitions from memory to the SG and noisy states takes place. In order to illustrate better the behavior of the system in the cases of interest, we also include in Figure 4 the time series of *m*^μ^(*t*) is some exemplary points for κ_∞_ = 20, as indicated in the phase diagrams by a pink star. Each panel corresponds to a given value of *P*, and each graph on them to a point in the (*T*, α) space. We find that the combination of thermal and quenched disorder, associated with the interference among patterns, can give rise to oscillations among the memorized patterns for α < 1—that is, when the networks are homogeneous—and *T* < 1, which are correspond to the yellow regions in the *g*_*p*_ panels of Figure 3. Note that the observed oscillations occur at level of the neuronal population as measured by the global network parameter *m*^μ^(*t*), and not on the single neuron level—which appear as small, high-frequency oscillations of *m*^μ^(*t*).

In order to illustrate the emergent behavior of the system, we refer here to Figure 3A, which corresponds to κ_∞_ = 20 and *P* = 5. The top graph in the panel represents *g*_*P*_, the number of patterns visited by the system after the transient evolution takes place. For α > 1, we find that this number remains finite, and greater than zero, up to very high values of the temperature (*T* ≈ 2.0) corresponding to the light-blue region in the top panel of Figure 3A, for instance. This indicates that the system is in a memory state (or in a SG-like state in which only a small number of patterns are retrieved), such as the ones depicted in the bottom graphs of Figure 4A. The stability of the memory state for *T* > 1 is possible due to the emergence of heterogeneous structures (since α > 1), and consequently hubs, as indicated in the black region of the bottom plot of Figure 3A, since *ḡ* → 0 for α > 1. Notice also in the middle graph of Figure 3A how, as *T* is increased, the overlap corresponding to these states, *m*_*P*_, decreases, indicating that these states are also becoming less stable as the thermal noise becomes stronger. In these conditions, only the more densely connected hub nodes are able to maintain information about the memories, and these are the ones contributing the most to the overlap.

As α is decreased, however, the behavior shown in the diagrams becomes more complex and different regions (phases) start to emerge. We find, as expected, that memory is completely lost for *T* ≫ 1, i.e., due to the strong noise the system falls into the noisy or non-memory state (as indicated respectively by the black region and by the dark-blue region of the top and middle diagrams of Figure 3A). In fact, now networks are homogeneous (α < 1) and there are no hubs that preserve memory (as indicated by *ḡ* → 1 in the bottom plot of Figure 3A, indicating that the degree distribution is homogeneous). A typical time series of *m*^μ^(*t*) for this situation is shown in the top-right graph of Figure 4B.

For small values of *T* and α (*T*, α < 1) on the other hand, *g*_*P*_ → 1 (orange and yellow region of the top panel of Figure 3A), indicating that a great number of patterns are being retrieved (*P*_*r*_ → *P*) with a moderate value of the overlap *m*_*P*_, as indicated by the green and blue region in the middle panel of Figure 3A. Moreover, results in Figure 2 indicate that, at least in some cases, *g*_*P*_ actually increases when *T* goes from 0 to 1 (see, e.g., the yellow area in the top panel of Figure 3A). That is, as the temperature increases, more memories take place in the state of the system, with a relatively high overlap *m*_*P*_. This is because, for α < 1, there is a wide region of oscillatory behavior between the SG-like and the noisy phases corresponding to the yellow region of the *g*_*p*_ diagrams in Figure 3. An exemplary series of oscillations is illustrated in the top-left graph of Figure 4A. This emerges as a consequence of the interplay between structural and thermal noise and the activity of network, since the process of addition and removal of synapses, that creates a dynamical network structure, together with the thermal noise, can make the recovered patterns unstable. Moreover, given that α < 1, the structure of the networks remains homogeneous and no real hubs emerge. Notice however that due to the non-trivial interplay between activity and topology, in the region of oscillatory behavior the networks display a more heterogeneous structure, and *ḡ* < 1. This effect will be discussed in more detail in the following section.

As in the previous section, we have also analyzed the role of the stationary mean connectivity, κ_∞_, on the phase diagram of the system for *T* > 0 (see Figure 3). This parameter holds physiological interests since it can be taken as a measure of the extension of the process of synaptic pruning. From this point of view, a brain that has undergone a more drastic synaptic pruning would have smaller κ_∞_ than one that has been less pruned. This is to be related to recent experiments that have associated an excessive pruning in certain brain areas with schizophrenia (Sekar et al., 2016), whereas ASD has been related to a defect of synaptic pruning (Tang et al., 2014). We find in our model that the area associated with the oscillatory behavior (for α, *T* < 1) for a given κ_∞_ is maximum at intermediate values of *P*: for very small *P* there is a dominance of stable SG-like states, whereas for large *P* the system falls easily on the noisy phase. Similarly, for a given *P* the greater extension of the oscillatory phase is found for an intermediate κ_∞_. For instance, for *P* = 10 the noisy phase extends to *T* < 1 for κ_∞_ = 20 (Figure 3B) and the oscillatory region is small, whereas for κ_∞_ = 60 (Figure 3J) there is a combination of stable SG-like states and oscillations for α, *T* < 1. Finally for κ_∞_ = 40 (Figure 3F) the oscillatory phase is most robust. Consequently, the absence of dynamical memories in the system could be associated with a defect of the pruning process that causes κ_∞_ to be greater than usual, and could be therefore associated with ASD. Interestingly, it has been recently reported that short-term memory and episodic memory are impaired in ASD subjects (Poirier et al., 2011; Lind et al., 2014), which is consistent with our findings here since, in order to be able to recall a sequence of memories, it is first necessary to destabilize the already recalled ones so as to allow the system to remember new ones. On the other hand, schizophrenia is typically associated with erratic behavior (Loh et al., 2007), which could be related to the high frequency memory oscillations found here for smaller values of κ_∞_.

### 3.3. Emergence of Hubs

The appearance of hubs and heterogeneity plays a significant role in the emergent dynamics of the system. In particular, with a given level of noise (*T* > 0), the topological structure of the network determines whether the system relaxes to a memory state, wanders among different patterns or falls into a noisy state. Therefore, here we discuss in more detail the emergence of hubs during the network evolution and their effect on the emergent state of the system.

We first notice that, according to the previous analysis, for α < 1 networks are homogeneous, as evidenced by the homogeneous degree distributions shown in the insets of Figures 2A,B. This is also revealed by the high value of the homogeneity parameter *ḡ* shown in Figures 1, 3 for α < 1, indicating that the variance of *k*_*i*_ is small. As a consequence, no real hubs can be defined, since all nodes have similar low degree (given that κ_∞_ ≪ *N*, so that the connectivity of the nodes is bounded). On the contrary, for α > 1 and in the case of memory, networks are heterogeneous as evidenced by *ḡ* → 0 (black regions in the corresponding diagrams *ḡ*(α, *P*) and *ḡ*(α, *T*) respectively in Figures 1, 3). This indicates that there are nodes with very different degrees and, in particular, the degree distribution *p*(*k, t*) is bimodal and it splits in two, as shown in the insets of Figure 2C,D, with the emergence of hubs. Therefore, one can set the connectivity threshold *k*_*th*_—that defines the minimum node's degree to characterize it as a hub—at the value of *k* at which *p*(*k, t* → ∞) presents a local minimum between the two modes. This establishes a clear separation between high and low degree nodes. In particular, in all cases studied here, we find that a threshold *k*_*th*_ = 2κ_∞_ also suffices to differentiate between homogeneous and heterogeneous structures, since for α < 1 (homogeneous case) the maximum degree of a network is always below 2κ_∞_.

Interestingly, due to the underlying stochastic rewiring process and to the system's finite size, there is always some variability in the degrees of the nodes and, particularly in the region of oscillatory behavior, there is a relative increase in the variability of *k*_*i*_ with respect to the SG phase (as evidence by a decreased *ḡ* in the corresponding diagrams of Figure 3). We argue that this is due to the intrinsic coupling between activity and topology, and to the combination of thermal (since *T* > 0), topological (due to the ongoing rewiring process) and quenched (due to the learning of different patterns) disorder in the system. In the region of oscillatory behavior, the instability of the memories influences the synaptic currents *I*_*i*_ creating variability, thus causing the observed increased heterogeneity. This causes the emergence of relatively-high degree nodes that correspond to the tail of the homogeneous distribution *p*(*k, t* → ∞) and which might have an important effect on the system. Therefore, in order to explore as well the dynamics of these relatively-high degree nodes, we have selected a lower threshold, *k*_*th*_ = 1.75κ_∞_, for the analysis of hub dynamics.

**Figure 5:**
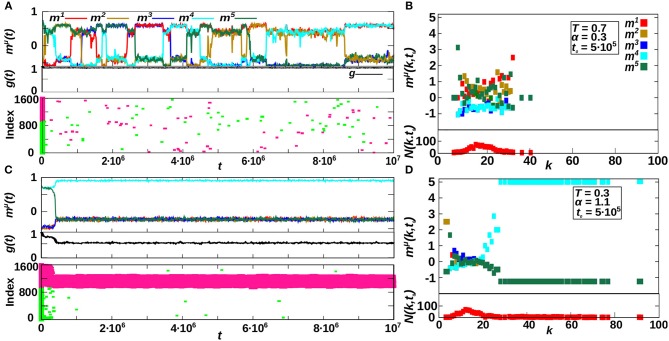
Emergence and effect of hubs in the system. **(A,C)** Show the temporal evolution of the system in two representative cases of the dynamics for *P* = 5 and κ_∞_ = 20, corresponding to the emergent oscillatory behavior for *T* = 0.7 and α = 0.3 **(A)** and to the heterogeneous memory phase for *T* = 1.1 and α = 1.1 **(C)**. In these panels, the top plots represent *m*^μ^(*t*), the middle ones the homogeneity parameter *g*(*t*), and finally the bottom ones show the existing hubs in the network at each time *t*, where active and inactive hubs are plotted in different colors (respectively, pink and green). **(B,D)** Show a snap-shot of the state of the system in **(A,C)**, respectively, at time $t_{0}=5\cdot 10^{5}$, as represented by the degree-dependent overlap $m^{\mu }\left(k,t_{0}\right)$ and the number of nodes with degree *k*, *N*(*k, t*_0_) = *Np*(*k, t*_0_). Results are for *N* = 1, 600.

Hubs (and relatively-high degree nodes for the homogeneous case) dynamics is investigated in Figure 5, where we compare two different cases for *P* = 5 and κ_∞_ = 20. The first one, shown in Figures 5A,B, corresponds to the region of oscillatory behavior for homogeneous networks (α < 1) and it is for *T* = 0.7 and α = 0.3, corresponding to the bottom-left graph of Figure 4A. The second one, shown in Figures 4C,D, corresponds to the heterogeneous-memory phase for α > 1, and it is for *T* = 0.3 and α = 1.1, corresponding to the top-left graph of Figure 4A. In particular, we analyze in Figures 4A,C the temporal evolution of the system as given by the overlap *m*^μ^(*t*), the homogeneity *g*(*t*) and the hub raster plots, where we represent the existing hubs at each time *t*, in different colors according to their active or inactive state (respectively pink and green). Furthermore, in Figures 4B,D we show the degree-dependent overlap $m^{\mu }\left(k,t_{0}\right)$ [defined in Equation (8)] and the degree histogram *N*(*k, t*_0_) = *Np*(*k, t*_0_), for a particular time, $t_{0}=5\cdot 10^{6}$MCS, corresponding to the systems respectively depicted in Figures 4A,C.

We observe, for α > 1 (Figure 5C), that a great number of hubs emerge in the system, and that almost all hubs correspond to the active nodes of the retrieved pattern. Moreover, in this case $m^{\mu }\left(k,t_{0}\right)$ of the recovered pattern μ is larger for high-degree nodes (Figure 5D), indicating that they contribute most to the overlap $m^{\mu }\left(t_{0}\right)$. On the contrary, for the non-recovered patterns ν, $m^{\nu }\left(k,t_{0}\right)$ remains small for all *k*. On the other hand, for α < 1, no real hubs emerge and only transient relatively-high degree nodes are observed in Figure 5A. These do not only correspond to the recovered patterns but are scattered throughout the network, and no significant correlation can be measured between the pattern oscillations and the hubs dynamics. This causes instabilities that ultimately lead to the oscillatory behavior (see Figure 5B, indicating that relatively high-degree nodes contribute more to *m*^μ^(*k, t*) of the recovered patterns but not only).

In summary, Figure 5 shows that for α > 1 there are active hubs in the system that correspond to the recovered pattern, making it stable. On the other hand, for α < 1 no real hubs can emerge in the system, and the transient relatively-high degree nodes are scattered throughout the network, not only corresponding to the recovered pattern, thus inducing the observed oscillatory behavior.

### 3.4. Analysis of the Oscillatory Behavior

**Figure 6:**
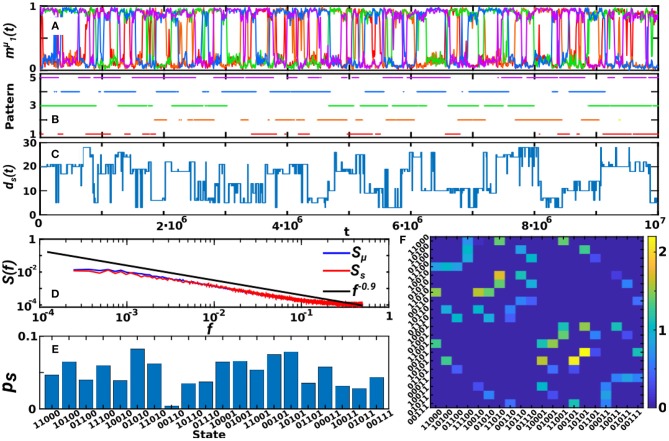
Analysis of the oscillatory behavior of the system in a representative point (*T* = 0.7, α = 0.3, *P* = 5, κ_∞_ = 20). **(A–C)** Show the temporal evolution of the system as given by the retrieved patterns at each time, $m_{1}^{\mu }\left(t\right)$ **(A)**, the binearized variable $m_{B}^{\mu }\left(t\right)$ indicating whether each pattern is active or not **(B)**, and finally the global memory state *d*_*s*_(*t*) **(C)** as defined in the text, for a total time of 10^7^ MCS. These show that the system wanders through the different attractors without a periodic order. **(D)** Shows the power spectra of $m_{B}^{\mu }\left(t\right)$, *S*_μ_(*f*), and of *d*_*s*_(*t*), *S*_*s*_(*f*), indicating a power-law scaling of *S*_μ_(*f*) and *S*_*s*_(*f*) with an exponent of −0.9. Finally, **(E)** shows the probability of appearance of each global state, evidencing that only 2− and 3−pattern SG states are recovered, and **(F)** shows the transition matrix of global states, that is, the probability of jumping (times 10^−3^) from a given global state *s* to another *s*′. Results are for *N* = 1, 600, and have been averaged over 20 realizations of the system in **(D,F)**.

In the previous sections we have shown the emergence of oscillations for α < 1 and *T* > 0 and their relation to the existence of transient relatively-high degree nodes on the network. Here, we develop further on the structure and patterns of these oscillations. For simplicity, we focus on the case of κ_∞_ = 20 and *P* = 5 as before, and in Figure 6 we show a long time series corresponding to this oscillatory phase (*T* = 0.7 and α = 0.3 as in the top graph of Figure 4A and in Figure 5A). Plots of the active-overlap parameter $m_{1}^{\mu }\left(t\right)$ (Figure 6A) defined in Equation (9), its binearized version $m_{B}^{\mu }\left(t\right)$ (Figure 6B) and the global memory state parameter *d*_*s*_(*t*) defined in Equation (10) (Figure 6C) indicate that the state of the system corresponds to oscillations between SG-like states in which either 2 or 3 patterns are transiently retrieved. These plots also evidence that the oscillations do not follow any clear periodic or regular pattern.

In order to analyze the pattern of oscillations, we show the power spectra of $m_{B}^{\mu }\left(t\right)$, *S*_μ_(*f*), and of *d*_*s*_(*t*), *S*_*s*_(*f*), in Figure 6F, which displays a power-law decay with an exponent equal to −0.9, indicating that there is not a dominant frequency of the oscillations, but that jumps between different patterns occur at all time scales. This is in accordance with previous studies that have repeatedly reported 1/*f*-type noise in brain activity under healthy conditions. It has been reported, for instance, in electroencephalogram (EEG) and functional magnetic resonance (fMRI) measures of human brain activity (Linkenkaer-Hansen et al., 2001; Voytek et al., 2015) and also in behavioral processes related to human cognition and motion as well as animal motion (Chialvo, 2010). 1/*f* noise indicates the existence of temporal correlations within the data, and has been related to emerging self-organized criticality in the brain (Chialvo, 2010).

Moreover, we also investigate the frequency of appearance of each global state, as seen in Figure 6E, which indicates that global states have different probabilities of occurrence in each realization of the system. However, when averaged over realizations, the mean probability of each state, ${\bar{p}}_{s}$, converges to a uniform distribution ${\bar{p}}_{s}\to 1/N_{s}$, where *N*_*s*_ = 20 is the total number of possible 2− and 3−pattern states (${\bar{p}}_{s}=0.054\left(3\right)$ when averaged over 20 realizations). Similarly, individual patterns may have different probability of appearance, *p*_μ_, in each realization of the dynamics, but when averaged over realizations ${\bar{p}}_{\mu }$ converges to 1/*P* [$\bar{p_{\mu }}=0.21\left(1\right)$]. Finally, we also computed the transition matrix between global SG-like states (Figure 6F) which indicates that, in a given realization of the system, some transitions are preferred by the system depending on the emergent coupling between activity and topology.

In summary, these results show that the oscillations are not periodic, but occur at all time scales, and that all SG-like states are visited in a non-periodic order. Interestingly however, in a given realization of the system not all transitions are allowed, but only some of them occur. It could be interesting to analyze in more detail in further studies whether the coupling between structure and activity induces a particular pattern of oscillations, and how the scaling of the frequency of oscillations depends on the parameters of model.

## 4. Discussion

We report here on recent studies of the emergent behavior of developing brain models in which structure and function cooperate and influence each other through a feedback loop, thus affecting the system's memory storage and retrieval abilities. This is a prominent example of how inter-synaptic factors at the network level can affect the processing of information in developing brains in a nontrivial way. In particular, our study focuses on the analysis of the conditions under which such feedback loop can enhance the storage and retrieval of a set of correlated patterns. Our work also pays attention to the emerging dynamics of the system, which is a consequence of the interplay between structural, quenched, and thermal disorder during its maturation. The results presented here demonstrate that a heterogeneous network can greatly improve the stability of the memory patterns, since its structure is optimized to preserve information about them in the network hubs which, as we have shown, correspond to the active neurons of the retrieved memories during the recall process. Moreover, due to the structural plasticity, once a pattern is retrieved, the ability of the system to recall it again increases thanks to pruning optimization. This illustrates the constructive role of synaptic pruning to consolidate memories in the “memory phase” of the system.

Our study also shows that the interplay between thermal noise, the interference among stored patterns and the dynamics driving the evolution of the topology creates instabilities on the memory attractors, which can make the system wander among different configurations for certain values of the parameters. In our model, these oscillations among stored patterns are caused by the topological synaptic plasticity due to the death and birth of synapses, which change the energy landscape of the system. In fact, in the absence of this rewiring process, this oscillatory phase is not present and the model would reduce to an Amari-Hopfield model on top of a non-trivial fixed topology. This has been shown to present the same phases as the canonical fully connected version of the model, with transition lines that depend on the topology, so that for instance the critical temperature diverges, *T*_*c*_ → ∞ as *N* → ∞, due to the presence of hubs that retain pattern information. Interestingly, in our model the oscillatory behavior that takes place on the homogeneous networks phase of the system is also associated with an increased transient heterogeneity of the underlying structure, in which transient relatively-high degree nodes (whose degree is however smaller than typical hubs) emerge and disappear in time. Moreover, these relatively-high degree nodes do not correspond in general to the active nodes of the transiently recovered patterns, but appear distributed throughout the whole network, corresponding also to active nodes of the rest of non retrieved patterns. This creates a non-trivial time-dependent competition among the different patterns which, together with the subsequent removal of some synapses during brain development, can make the currently recalled attractor less stable, thus inducing the observed wandering among the memories.

We have also analyzed the characteristics of the oscillations and shown that the oscillatory pattern is not periodic but presents a power spectrum following a power law scaling decay with an exponent of −0.9, so there are not any preferred frequencies. This in accordance with previous studies repeatedly reporting 1/*f* noise in brain activity under healthy conditions (Linkenkaer-Hansen et al., 2001; Voytek et al., 2015) and also in behavioral processes (Chialvo, 2010), which is related to the existence of temporal correlations within the data, and has been related to emerging self-organized criticality (SOC) in the brain (Chialvo, 2010).

Interestingly, the appearance of an oscillatory phase characterized by dynamical memories could be useful to enhance the learning and recalling of sequences of patterns of activity, as in episodic memories, without the necessity of any external input or current forcing the retrieval of the memories in the sequence. This type of oscillations has already been reported for brain models with synapses enduring short-term synaptic plasticity (STP) (Pantic et al., 2002; Cortés et al., 2006; Marro et al., 2007; Torres et al., 2007, 2008). This occurs at the synapse level and depends on the activity of the pre-synaptic neuron (which therefore depends closely on the synaptic current *I*_*i*_ used in our model Amit, 1989). However, STP is caused by biophysical mechanisms controlling the release and recycling of neurotransmitters at the synapses during synaptic transmission and operates at short time scales of the order of *ms* (Tsodyks and Markram, 1997). The activity dependent topological plasticity reported here, however, is the result of the interplay between form and function in a developing brain, and the ongoing synaptic rewiring in mature brains, which happens at the time scale of hours or days (Holtmaat and Svoboda, 2009). Moreover, topological plasticity allows the system to explore more efficiently its dynamical phase space and it has been shown to improve the capacity of neural networks by allowing them to organize in a more efficient structure. Both mechanisms could happen at the same time in actual systems, together with neuron level phenomena such as spike adaptation (Knoblauch and Palm, 2002; Ha and Cheong, 2017). We hypothesize that the combination of these mechanisms could lead to the extension of the oscillatory behavior to other regions of the phase diagram, although results would strongly depend on the relative time scale between structural plasticity and STP, and it could be an interesting approach for future works.

It is also worth noting that the reported oscillations in our system are for the overlap function that is a measure of the activity of the whole neuron population during memory recall processes. These occur in actual neural systems at a long time scale—normally days or even years—as it is the case in our model. Temporal changes at the single neuron level appear in our system as high frequency fluctuations in the time dependent value of the overlap parameter. If the level of stochasticity is low (low *T*) and the network size is large enough (*N* ≫ 1), such single neuron fluctuations are very unlike to be significant on *m*^μ^(*t*). In any case, the model output could be easily tuned up to obtain faster of slower oscillations in the overlap function to match more realistically actual experiments during learning and recalling. This could be done by varying some model parameters to make the recall process more or less efficient in time, or to allow the system to recall dynamic memories—such as episodic memories—that are learned and recalled at different stimuli input frequencies.

We have analyzed in detail how the dynamical behavior of the system depends on the synaptic factors affecting the addition and removal of synapses and on the number of stored patterns. In particular, the stationary mean connectivity of the network, κ_∞_, has been shown to have a great effect on the emergent behavior of the system. For instance, we have found that the absence of dynamical memories in the system, or the presence of memory oscillations with long periods, is associated with a defect of the pruning process. Similarly, we have shown that high frequency oscillations among patterns and more tendency to noisy behavior occur when there is a pruning excess. In particular, the destabilization of recovered memories is necessary for instance to recall a sequence of memories, each during a short period of time, so as to allow the system to remember new ones. One may argue that the induced instability and the associated oscillatory behavior observed in our system could be positive for information processing, since it would allow neuronal media to explore different memories or attractors, for instance following hetero-clinic orbits, and consequently, to process more complex information, such as spatio-temporal patterns of information (see Rabinovich et al., 2006 and references therein). Such emergent behavior could also be useful to respond more efficiently to changing external stimuli, as in episodic memory tasks, as it has been widely stated in previous works in different neural systems (Cortés et al., 2006; Marro et al., 2008; Torres et al., 2008).

The different emergent behavior in the model with varying connectivity could perhaps be associated with cognitive abilities related to autism spectrum disorders (ASD) and schizophrenia. In the former, a pruning defect has been observed in some brain areas (Tang et al., 2014), and it has recently been reported that short-term memory and episodic memory are impaired in ASD subjects (Poirier et al., 2011; Lind et al., 2014). Our results are consistent with these observations since, if the brain is less pruned, the mean connectivity of the corresponding network is higher, thus making the memory attractors more stable. This implies a lower ability of the brain to remember sequences of patterns as described in episodic memory tasks in ASD patients because it is harder for the brain to forget the already recalled pattern due to its strong stability. Results in our model indicate that a lightly pruned brain could be forced out of the memory phase into an oscillatory regime with an increase in the number of stored patterns (see Figure 3). These observations might provide an interesting insight for experimental psychologists to design a cognitive strategy or therapy to learn and recall sequences of patterns, that might improve the cognitive abilities of patients with ASD. On the other hand, we have demonstrated that high frequency oscillations among patterns occur when there is a pruning excess, and this could be perhaps associated with the erratic behavior observed in schizophrenia (Loh et al., 2007), in which case the brain seems to present some areas with an excess of pruning (Sekar et al., 2016). In this case our results here suggest that a learning therapy based on increasing the number of stored memories would not be useful but may in fact be detrimental, as it would make the memory activity patterns more unstable. A learning therapy that moves the patient brain state near to its stable memory phase, for instance, by stabilizing a few old useful memories, could therefore be more convenient.

Finally, we note also that some drastic assumptions have been made in order to simplify the relevant scenario. Firstly, our study is for sparse correlated patterns, as suggested by experimental studies (Chklovskii et al., 2004; Akam and Kullmann, 2014), which are also known to improve the memory retrieval capabilities of the network (Knoblauch et al., 2014; Knoblauch and Sommer, 2016) and particularly so in the case of highly sparse and heterogeneous networks (Morelli et al., 2004). Moreover, we have selected the patterns of activity to be non-overlapping regions of activity, following previous works (Torres and Marro, 2015). This set up corresponds to a particular case that allows for a better visualization of the network dynamics and that has proven out to be useful to investigate the interplay between structure and dynamics, i.e., between form and function, together with the presence of thermal and quenched disorder, on a developing neural network. Similarly, results are for the low storage regime of the neural network, *P* ≪ *N*, what allows us to study in detail the dynamical behavior of the system that gives rise to memory wandering. However, given that our qualitative results depend little on *P* for *P* > 20, we expect them to hold when *P* is increased.

Further extensions of this work could also include the consideration of different details of the synaptic pruning process, including for instance the growth of synapses taking place after birth (Millán et al., 2018b), multiple synaptic contacts between neurons (Knoblauch et al., 2014; Knoblauch and Sommer, 2016), or a hard bound on the maximum degree of the nodes (Stepanyants et al., 2002; Fares and Stepanyants, 2009). Moreover, more elaborated definitions of the probabilities of growth and death of synapses (Equations 16 and 18) could also be considered, such as a mechanism of self-organization toward the stationary mean connectivity (Chechik et al., 1999; de Arcangelis et al., 2006; Lewis and Todd, 2007; Tetzlaff et al., 2010) or by explicitly including a dynamics for the available nutrients (Tetzlaff et al., 2010). However these definitions would still need to reproduce the basic characteristics of brain development and synaptic pruning, that is, an initial fast decay of connectivity and an ongoing rewiring of edges after the stationary mean connectivity has been reached. We expect that our main results (existence of a feed-back loop between structure and activity, bistability, and emergence of oscillations) would still hold, at least qualitatively, with these modifications, in accordance with previous studies (Millán et al., 2018b). Similarly, the local probabilities in Equation (16) could also consider more detailed functions, to characterize for instance a specific dependence on the concentration of different proteins and growth factors controlling synaptic growth. These could be obtained experimentally, although to the best of our knowledge it has not yet been done. Our work could thus motivate neurobiologists to design experiments to describe the exact probabilities involved in synaptogenesis and pruning, information that could be easily incorporated in our theoretical framework. Similarly, it could also be particularly interesting to include a learning dynamics that is also coupled to the development of the neural network, thus modeling learning during infancy, or to include an external current on the system, that could certainly be time dependent, to analyze the effect of external inputs on associative memory and memory wandering.

## Author Contributions

APM, JJT and JM designed the analyses, discussed the results, and wrote the manuscript. APM also wrote the codes and performed the simulations.

### Conflict of Interest Statement

The authors declare that the research was conducted in the absence of any commercial or financial relationships that could be construed as a potential conflict of interest.

## References

[B1] Abbott L. LeMasson G. (1993). Analysis of neuron models with dynamically regulated conductances. Neural Comput. 5, 823–842. 10.1162/neco.1993.5.6.823

[B2] Akam T. Kullmann D. M. (2014). Oscillatory multiplexing of population codes for selective communication in the mammalian brain. Nat. Rev. Neurosci. 15, 111–122. 10.1038/nrn3668 24434912 PMC4724886

[B3] Albert R. (2005). Scale-free networks in cell biology. J. Cell Sci. 118, 4947–4957. 10.1242/jcs.02714 16254242

[B4] Amari S.-I. (1972). Characteristics of random nets of analog neuron-like elements. IEEE Trans. Syst. Man Cybern. 2, 643–657.

[B5] Amit D. J. (1989). Modeling Brain Function: the World of Attractor Neural Networks. New York, NY: Cambridge University Press.

[B6] Azouz R. Gray C. M. (2000). Dynamic spike threshold reveals a mechanism for synaptic coincidence detection in cortical neurons *in vivo*. Proc. Natl. Acad. Sci. U.S.A. 97, 8110–8115. 10.1073/pnas.130200797 10859358 PMC16678

[B7] Azouz R. Gray C. M. (2003). Adaptive coincidence detection and dynamic gain control in visual cortical neurons *in vivo*. Neuron 37, 513–523. 10.1016/S0896-6273(02)01186-8 12575957

[B8] Boccaletti S. Latora V. Moreno Y. Chavez M. Hwang D.-U. (2006). Complex networks: structure and dynamics. Phys. Rep. 424, 175–308. 10.1016/j.physrep.2005.10.009

[B9] Bortz A. B. Kalos M. H. Lebowitz J. L. (1975). A new algorithm for monte carlo simulation of ising spin systems. J. Comput. Phys. 17, 10–18. 10.1016/0021-9991(75)90060-1

[B10] Bullmore E. Sporns O. (2009). Complex brain networks: graph theoretical analysis of structural and functional systems. Nat. Rev. Neurosci. 10, 186–198. 10.1038/nrn2575 19190637

[B11] Cardin J. A. Palmer L. A. Contreras D. (2008). Cellular mechanisms underlying stimulus-dependent gain modulation in primary visual cortex neurons *in vivo*. Neuron 59, 150–160. 10.1016/j.neuron.2008.05.002 18614036 PMC2504695

[B12] Castillo I. P. Wemmenhove B. Hatchett J. Coolen A. Skantzos N. Nikoletopoulos T. (2004). Analytic solution of attractor neural networks on scale-free graphs. J. Phys. A Math. Gen. 37:8789 10.1088/0305-4470/37/37/002

[B13] Chechik G. Meilijson I. Ruppin E. (1998). Synaptic pruning in development: a computational account. Neural Comput. 10, 1759–1777. 10.1162/089976698300017124 9744896

[B14] Chechik G. Meilijson I. Ruppin E. (1999). Neuronal regulation: a mechanism for synaptic pruning during brain maturation. Neural Comput. 11, 2061–2080. 10.1162/089976699300016089 10578044

[B15] Chialvo D. R. (2010). Emergent complex neural dynamics. Nat. Phys. 6, 744–750. 10.1038/nphys1803

[B16] Chklovskii D. B. Mel B. Svoboda K. (2004). Cortical rewiring and information storage. Nature 431, 782–788. 10.1038/nature03012 15483599

[B17] Cortés J. M. Torres J. J. Marro J. Garrido P. L. Kappen H. J. (2006). Effects of fast presynaptic noise in attractor neural networks. Neural Comput. 18, 614–633. 10.1162/neco.2006.18.3.614 16483410

[B18] Crossley N. A. Mechelli A. Scott J. Carletti F. Fox P. T. McGuire P.. (2014). The hubs of the human connectome are generally implicated in the anatomy of brain disorders. Brain 137, 2382–2395. 10.1093/brain/awu132 25057133 PMC4107735

[B19] de Arcangelis L. Perrone-Capano C. Herrmann H. J. (2006). Self-organized criticality model for brain plasticity. Phys. Rev. Lett. 96:028107. 10.1103/PhysRevLett.96.028107 16486652

[B20] Faludi G. Mirnics K. (2011). Synaptic changes in the brain of subjects with schizophrenia. Int. J. Dev. Neurosci. 29, 305–309. 10.1016/j.ijdevneu.2011.02.013 21382468 PMC3074034

[B21] Fares T. Stepanyants A. (2009). Cooperative synapse formation in the neocortex. Proc. Natl. Acad. Sci. U.S.A. 106, 16463–16468. 10.1073/pnas.0813265106 19805321 PMC2738618

[B22] Fornito A. Zalesky A. Breakspear M. (2015). The connectomics of brain disorders. Nat. Rev. Neurosci. 16, 159–172. 10.1038/nrn3901 25697159

[B23] Fricker D. Verheugen J. A. Miles R. (1999). Cell-attached measurements of the firing threshold of rat hippocampal neurones. J. Physiol. 517, 791–804. 10.1111/j.1469-7793.1999.0791s.x 10358119 PMC2269376

[B24] Gastner M. T. Ódor G. (2016). The topology of large open connectome networks for the human brain. Sci. Rep. 6:27249. 10.1038/srep27249 27270602 PMC4895133

[B25] Geschwind D. H. Levitt P. (2007). Autism spectrum disorders: developmental disconnection syndromes. Curr. Opin. Neurobiol. 17, 103–111. 10.1016/j.conb.2007.01.009 17275283

[B26] Gross T. Blasius B. (2008). Adaptive coevolutionary networks: a review. J. R. Soc. Interface 5, 259–271. 10.1098/rsif.2007.1229 17971320 PMC2405905

[B27] Ha G. E. Cheong E. (2017). Spike frequency adaptation in neurons of the central nervous system. Exp. Neurobiol. 26, 179–185. 10.5607/en.2017.26.4.179 28912640 PMC5597548

[B28] Hobbiss A. F. Ramiro-Cortés Y. Israely I. (2018). Homeostatic plasticity scales dendritic spine volumes and changes the threshold and specificity of hebbian plasticity. iScience 8, 161–174. 10.1016/j.isci.2018.09.015 30317078 PMC6187013

[B29] Holtmaat A. Svoboda K. (2009). Experience-dependent structural synaptic plasticity in the mammalian brain. Nat. Rev. Neurosci. 10, 647–658. 10.1038/nrn2699 19693029

[B30] Hopfield J. J. (1982). Neural networks and physical systems with emergent collective computational abilities. Proc. Natl. Acad. Sci. U.S.A. 79, 2554–2558. 10.1073/pnas.79.8.2554 6953413 PMC346238

[B31] Horn D. Usher M. (1989). Neural networks with dynamical thresholds. Phys. Rev. A 40:1036. 10.1103/PhysRevA.40.1036 9902229

[B32] Huttenlocher P. R. Dabholkar A. S. (1997). Regional differences in synaptogenesis in human cerebral cortex. J. Compar. Neurol. 387, 167–178. 10.1002/(SICI)1096-9861(19971020)387:2<167::AID-CNE1>3.0.CO;2-Z 9336221

[B33] Iglesias J. Eriksson J. Grize F. Tomassini M. Villa A. E. (2005). Dynamics of pruning in simulated large-scale spiking neural networks. Biosystems 79, 11–20. 10.1016/j.biosystems.2004.09.016 15649585

[B34] Itskov V. Curto C. Pastalkova E. Buzsáki G. (2011). Cell assembly sequences arising from spike threshold adaptation keep track of time in the hippocampus. J. Neurosci. 31, 2828–2834. 10.1523/JNEUROSCI.3773-10.2011 21414904 PMC3097063

[B35] Johnson S. Marro J. Torres J. J. (2010). Evolving networks and the development of neural systems. J. Stat. Mech. 2010:P03003 10.1088/1742-5468/2010/03/P03003

[B36] Keshavan M. S. Anderson S. Pettergrew J. W. (1994). Is schizophrenia due to excessive synaptic pruning in the prefrontal cortex? The feinberg hypothesis revisited. J. Psychiatr. Res. 28, 239–265. 10.1016/0022-3956(94)90009-4 7932285

[B37] Klintsova A. Y. Greenough W. T. (1999). Synaptic plasticity in cortical systems. Curr. Opin. Neurobiol. 9, 203–208. 10.1016/S0959-4388(99)80028-2 10322189

[B38] Knoblauch A. Körner E. Körner U. Sommer F. T. (2014). Structural synaptic plasticity has high memory capacity and can explain graded amnesia, catastrophic forgetting, and the spacing effect. PLoS ONE 9:e96485. 10.1371/journal.pone.0096485 24858841 PMC4032253

[B39] Knoblauch A. Palm G. (2002). Scene segmentation by spike synchronization in reciprocally connected visual areas. I. Local effects of cortical feedback. Biol. Cybern. 87, 151–167. 10.1007/s00422-002-0331-4 12200612

[B40] Knoblauch A. Palm G. Sommer F. T. (2010). Memory capacities for synaptic and structural plasticity. Neural Comput. 22, 289–341. 10.1162/neco.2009.08-07-588 19925281

[B41] Knoblauch A. Sommer F. T. (2016). Structural plasticity, effectual connectivity, and memory in cortex. Front. Neuroanat. 10:63. 10.3389/fnana.2016.00063 27378861 PMC4909771

[B42] Kobayashi R. Tsubo Y. Shinomoto S. (2009). Made-to-order spiking neuron model equipped with a multi-timescale adaptive threshold. Front. Comput. Neurosci. 3:9. 10.3389/neuro.10.009.2009 19668702 PMC2722979

[B43] Kolb B. Mychasiuk R. Muhammad A. Li Y. Frost D. O. Gibb R. (2012). Experience and the developing prefrontal cortex. Proc. Natl. Acad. Sci. U.S.A. 109(Suppl. 2), 17186–17193. 10.1073/pnas.1121251109 23045653 PMC3477383

[B44] Lewis M. D. Todd R. M. (2007). The self-regulating brain: cortical-subcortical feedback and the development of intelligent action. Cogn. Dev. 22, 406–430. 10.1016/j.cogdev.2007.08.004

[B45] Lind S. E. Williams D. M. Bowler D. M. Peel A. (2014). Episodic memory and episodic future thinking impairments in high-functioning autism spectrum disorder: an underlying difficulty with scene construction or self-projection? Neuropsychology 28, 55–67. 10.1037/neu0000005 24015827 PMC3906795

[B46] Linkenkaer-Hansen K. Nikouline V. V. Palva J. M. Ilmoniemi R. J. (2001). Long-range temporal correlations and scaling behavior in human brain oscillations. J. Neurosci. 21, 1370–1377. 10.1523/JNEUROSCI.21-04-01370.2001 11160408 PMC6762238

[B47] Loh M. Rolls E. T. Deco G. (2007). A dynamical systems hypothesis of schizophrenia. PLoS Comput. Biol. 3, 1–11. 10.1371/journal.pcbi.0030228 17997599 PMC2065887

[B48] Marro J. Torres J. J. Cortés J. M. (2007). Chaotic hopping between attractors in neural networks. Neural Netw. 20, 230–235. 10.1016/j.neunet.2006.11.005 17196366

[B49] Marro J. Torres J. J. Cortes J. M. (2008). Complex behavior in a network with time-dependent connections and silent nodes. J. Stat. Mech. 2008:P02017 10.1088/1742-5468/2008/02/P02017

[B50] Mejias J. F. Torres J. J. (2011). Emergence of resonances in neural systems: the interplay between adaptive threshold and short-term synaptic plasticity. PLoS ONE 6:e17255. 10.1371/journal.pone.0017255 21408148 PMC3050837

[B51] Millán A. P. Torres J. J. Johnson S. Marro J. (2018a). Concurrence of form and function in developing networks and its role in synaptic pruning. Nat. Commun. 9:2236. 10.1038/s41467-018-04537-6 29884799 PMC5993834

[B52] Millán A. P. Torres J. J. Johnson S. Marro J. (2018b). Growth strategy determines network performance. arXiv:1806.01878.

[B53] Morelli L. G. Abramson G. Kuperman M. N. (2004). Associative memory on a small-world neural network. Eur. Phys. J. B 38, 495–500. 10.1140/epjb/e2004-00144-7

[B54] Navlakha S. Barth A. L. Bar-Joseph Z. (2015). Decreasing-rate pruning optimizes the construction of efficient and robust distributed networks. PLoS Comput. Biol. 11, 1–23. 10.1371/journal.pcbi.1004347 26217933 PMC4517947

[B55] Oh S. W. Harris J. A. Ng L. Winslow B. Cain N. Mihalas S.. (2014). A mesoscale connectome of the mouse brain. Nature 508, 207–217. 10.1038/nature13186 24695228 PMC5102064

[B56] Oshima H. Odagaki T. (2007). Storage capacity and retrieval time of small-world neural networks. Phys. Rev. E 76:036114. 10.1103/PhysRevE.76.036114 17930313

[B57] Pantic L. Torres J. J. Kappen H. J. Gielen S. C. A. M. (2002). Associative memory with dynamic synapses. Neural Comput. 14, 2903–2923. 10.1162/089976602760805331 12487797

[B58] Poirier M. Martin J. S. Gaigg S. B. Bowler D. M. (2011). Short-term memory in autism spectrum disorder. J. Abnorm. Psychol. 120, 247–252. 10.1037/a0022298 21319933

[B59] Presumey J. Bialas A. R. Carroll M. C. (2017). Complement system in neural synapse elimination in development and disease. Adv. Immunol. 135, 53–79. 10.1016/bs.ai.2017.06.004 28826529

[B60] Rabinovich M. I. Varona P. Selverston A. I. Abarbanel H. D. (2006). Dynamical principles in neuroscience. Rev. Modern Phys. 78:1213 10.1103/RevModPhys.78.1213

[B61] Santos E. Noggle C. A. (2011). Synaptic pruning, in Encyclopedia of Child Behavior and Development, eds Goldstein S. Naglieri J. A. (Boston, MA: Springer US), 1464–1465.

[B62] Sayama H. Pestov I. Schmidt J. Bush B. J. Wong C. Yamanoi J. (2013). Modeling complex systems with adaptive networks. Comput. Math. Appl. 65, 1645–1664. 10.1016/j.camwa.2012.12.005

[B63] Sekar A. Bialas A. R. de Rivera H. Davis A. Hammond T. R. Kamitaki N.. (2016). Schizophrenia risk from complex variation of complement component 4. Nature 530, 177–183. 10.1038/nature16549 26814963 PMC4752392

[B64] Sompolinsky H. Kanter I. (1986). Temporal association in asymmetric neural networks. Phys. Rev. Lett. 57, 2861–2864. 10.1103/PhysRevLett.57.2861 10033885

[B65] Stafford J. M. Jarrett B. R. Miranda-Dominguez O. Mills B. D. Cain N. Mihalas S.. (2014). Large-scale topology and the default mode network in the mouse connectome. Proc. Natl. Acad. Sci. U.S.A. 111, 18745–18750. 10.1073/pnas.1404346111 25512496 PMC4284535

[B66] Stauffer D. Aharony A. da Fontoura Costa L. Adler J. (2003). Efficient hopfield pattern recognition on a scale-free neural network. Eur. Phys. J. B 32, 395–399. 10.1140/epjb/e2003-00114-7

[B67] Stepanyants A. Hof P. R. Chklovskii D. B. (2002). Geometry and structural plasticity of synaptic connectivity. Neuron 34, 275–288. 10.1016/S0896-6273(02)00652-9 11970869

[B68] Tang G. Gudsnuk K. Kuo S.-H. Cotrina M. L. Rosoklija G. Sosunov A.. (2014). Loss of mtor-dependent macroautophagy causes autistic-like synaptic pruning deficits. Neuron 83, 1131–1143. 10.1016/j.neuron.2014.07.040 25155956 PMC4159743

[B69] Tetzlaff C. Okujeni S. Egert U. Wörgötter F. Butz M. (2010). Self-organized criticality in developing neuronal networks. PLoS Comput. Biol. 6:e1001013. 10.1371/journal.pcbi.1001013 21152008 PMC2996321

[B70] Torres J. J. Cortes J. M. Marro J. Kappen H. J. (2007). Competition between synaptic depression and facilitation in attractor neural networks. Neural Comput. 19, 2739–2755. 10.1162/neco.2007.19.10.2739 17716010

[B71] Torres J. J. Marro J. (2015). Brain performance versus phase transitions. Sci. Rep. 5:12216. 10.1038/srep12216 26193453 PMC4507401

[B72] Torres J. J. Marro J. Cortes J. M. Wemmenhove B. (2008). Instabilities in attractor networks with fast synaptic fluctuations and partial updating of the neurons activity. Neural Netw. 21, 1272–1277. 10.1016/j.neunet.2008.07.002 18701255

[B73] Torres J. J. Muñoz M. A. Marro J. Garrido P. L. (2004). Influence of topology on the performance of a neural network. Neurocomputing 58, 229–234. 10.1016/j.neucom.2004.01.048

[B74] Tsodyks M. V. Markram H. (1997). The neural code between neocortical pyramidal neurons depends on neurotransmitter release probability. Proc. Natl. Acad. Sci. U.S.A. 94, 719–723. 10.1073/pnas.94.2.719 9012851 PMC19580

[B75] Turrigiano G. G. Leslie K. R. Desai N. S. Rutherford L. C. Nelson S. B. (1998). Activity-dependent scaling of quantal amplitude in neocortical neurons. Nature 391, 892–896. 10.1038/36103 9495341

[B76] Uhlig M. Levina A. Geisel T. Herrmann M. (2013). Critical dynamics in associative memory networks. Front. Comput. Neurosci. 7:87. 10.3389/fncom.2013.00087 23898261 PMC3721048

[B77] Van Den Heuvel M. P. Sporns O. (2011). Rich-club organization of the human connectome. J. Neurosci. 31, 15775–15786. 10.1523/JNEUROSCI.3539-11.2011 22049421 PMC6623027

[B78] Vazquez F. Eguíluz V. M. Miguel M. S. (2008). Generic absorbing transition in coevolution dynamics. Phys. Rev. Lett. 100:108702. 10.1103/PhysRevLett.100.108702 18352241

[B79] Voytek B. Kramer M. A. Case J. Lepage K. Q. Tempesta Z. R. Knight R. T.. (2015). Age-related changes in 1/f neural electrophysiological noise. J. Neurosci. 35, 13257–13265. 10.1523/JNEUROSCI.2332-14.2015 26400953 PMC4579381

